# The Ethanolic Extract of Lindera aggregata Modulates Gut Microbiota Dysbiosis and Alleviates Ethanol-Induced Acute Liver Inflammation and Oxidative Stress SIRT1/Nrf2/NF-*κ*B Pathway

**DOI:** 10.1155/2022/6256450

**Published:** 2022-12-20

**Authors:** Xin Zhao, MingMing Tan, YiChen He, Yi Pan, XinLi Fan, LuYi Ye, LongCai Liu, LuoQin Fu

**Affiliations:** ^1^Hepatobiliary and Pancreatic Surgery, Key Laboratory of Tumor Molecular Diagnosis and Individualized Medicine of Zhejiang Province, Zhejiang Provincial People's Hospital, Affiliated People's Hospital, Hangzhou Medical College, Hangzhou, Zhejiang 310014, China; ^2^College of Pharmacy, Hangzhou Medical College, Hangzhou 310059, China; ^3^Clinical Research Institute, Key Laboratory of Tumor Molecular Diagnosis and Individualized Medicine of Zhejiang Province, Zhejiang Provincial People's Hospital, Affiliated Hospital of Hangzhou Medical College, Hangzhou 310014, China; ^4^Emergency Department, People's Hospital of Tiantai County, Tiantai, Zhejiang 317200, China; ^5^College of Pharmacy, Zhejiang University of Technology, Hangzhou 310014, China; ^6^Department of Basic Medicine and Forensic Medicine, Hangzhou Medical College, Hangzhou 310059, China

## Abstract

This study is an attempt to evaluate the therapeutic effect of the ethanolic extract of *Lindera aggregata* on the liver and intestinal microbiota in rats with alcohol-induced liver injury (ALI). Rats were treated with 70 mg probiotics, 1 g/kg, 2 g/kg, and 3 g/kg ethanolic extract of *Lindera aggregata*, respectively, for 10 days. We found that *Lindera aggregata* could significantly reduce the biochemical parameters in the serum of ALD rats. *Lindera aggregata* alleviates oxidative stress and inflammation by upregulating SIRT1 and Nrf2 and downregulating COX2 and NF-*κ*B. The results of 16S rRNA gene sequencing showed that the medium dose of *Lindera aggregata* had the best effect on the growth of beneficial bacteria. Diversity analysis and LEfSe analysis showed that beneficial bacteria gradually occupied the dominant niche. The relative abundance of potential pathogens in the gut decreased significantly. We demonstrated that the ethanolic extract of *Lindera aggregata* can alleviate the oxidative stress and inflammation induced by alcohol through the SIRT1/Nrf2/NF-*κ*B pathway and can modulate the disturbance of gut microbiota induced by alcohol intake.

## 1. Introduction

Alcohol-induced liver injury (ALI) is still a clinical challenge with no effective treatment strategy. Alcohol-induced progressive damage begins with the accumulation of triglycerides in liver cells, and 10-35% of people develop further inflammation of the liver and liver cell damage and persistent liver injury, and inflammation eventually leads to progressive fibrosis and cirrhosis, which can eventually lead to the development of hepatocellular carcinoma [1, 2]. This suggests that novel and effective therapeutic drugs need to be developed and that the ethanol extract of *Lindera aggregata* has therapeutic potential for ALI.

Various pathways are involved in the pathogenesis of ALI. Increased oxidative stress and persistent high levels of inflammation play a key role in the development of ALI. Recently, the SIRT1/Nrf2/NF-*κ*B pathway has been found to play an important role in the signal transduction of oxidative stress and inflammation [3, 4]. Redox imbalance causes lipid peroxidation, which causes cells to lose their antioxidant defenses, resulting in dynamic imbalances in the antioxidant system in ALI that targets the antioxidant regulatory gene Nrf2 [5]. The increased reactive oxygen species (ROS) induced by ethanol exposure also interfered with the nuclear-cytoplasmic shuttle of SIRT1 and finally inhibited the activity of SIRT1 deacetylase in the liver, while the decrease in the level of SIRT1 level caused further liver injury and expression of proinflammatory cytokine mRNA expression [6, 7]. Meanwhile, SIRT1 deficiency leads to high acetylation of NF-*κ*B. Activated NF-*κ*B regulates the transcription and translation of multiple genes including inflammatory factors and chemokines by upregulating the expression of COX2, which further leads to a high and persistent expression of liver inflammation [8, 9]. Therefore, scavenging free radicals in the liver, balancing the antioxidant system, and reducing the expression of inflammatory factors are necessary measures to protect the liver from damage.

In recent years, the use of traditional Chinese medicine and natural products, with a lot of active components, multitarget action, and less toxicity, for the improvement of alcoholic liver disease has attracted the attention of an increasing number of researchers [10]. *Lindera aggregata* is the dried root of *Lindera aggregata*, a plant of Lauraceae. *Lindera aggregata* has been shown to have antitumor [11], antiproline endopeptidase [12], anti-inflammatory, and analgesic effects [13]. The total alkaloid of *Lindera aggregata* may inhibit the proliferation and activation of lymphocytes and reduce the inflammatory reaction in rheumatoid arthritis mice; meanwhile [14], NF-*κ*B signal pathways are blocked to inhibit the production of inflammatory mediators by macrophages [15].

In addition, the gut microbiota plays a role in the pathogenesis of alcohol-mediated liver disease. The gut microbiota is involved in the process of pathogenic bacteria, regulation of barrier function [16, 17], and liver and systemic inflammation through various mechanisms and maintains host intestinal homeostasis [18]. The evidence suggests that alcohol-induced changes in the gut microbiota contribute to the development of ALI [19]. Microbial dysbiosis is not only the result of alcohol intake but also directly related to the severity of ALI and individual susceptibility [20]. Some studies have shown that probiotics can reverse liver damage [21, 22]. More studies are needed to demonstrate the role of the gut microbiota in the development of alcohol-mediated liver injury. We used clinically approved probiotic adjuvant as a control, through the comparison to further illustrate the possible role of *Lindera aggregata*.

Studies have previously shown that *Lindera aggregata* has a better therapeutic effect on alcoholic liver disease, and the alcohol extract is better than the water extract, and the alcohol extract contains a higher content of active ingredient. The short-term intervention using edible alcohol is a simple and proven effective way to construct an alcoholic liver injury model [23]. The inflammatory burst and oxidative stress in the mouse brought about by alcohol mimics the state of alcoholic liver injury extremely well [24]. We further demonstrated the therapeutic value of *Lindera aggregata* through this model and examined the changes in the gut microbes of the mouse in this model. In this study, the regulation mechanism of *Lindera aggregata* on improving alcoholic liver injury was elucidated from the aspects of inhibiting inflammation and antioxidation and improving the structure of microflora at the whole and molecular levels; it provides a new choice of traditional Chinese medicine for the treatment of alcohol-induced acute liver injury and a basis for the development of new monomeric drugs for the effective treatment of alcohol-induced liver injury in *Lindera aggregata*.

## 2. Materials and Methods

### 2.1. Extraction and Quality Control of *Lindera aggregata*

*Lindera aggregata* was extracted with 12 BV 75% ethanol for 1.5 h, and the drug residue was extracted with 10 BV ethanol for 1 h. Collect all the liquid medicine, decompress to recover the ethanol, and concentrate it properly. Finally, the ethanol extract of *Lindera aggregata* was obtained. Before use, properly dilute with double distilled water, so that each milliliter of liquid medicine is equivalent to 0.5 g of crude drugs.

### 2.2. Establishment of Rat Model of Alcoholic Liver Injury

36 healthy male Sprague Dawley rats were randomly assigned to the blank control group (*n* = 6), model group (*n* = 6), BIFICO group (positive control) (*n* = 6), low-dose group (LGL, *n* = 6), middle-dose group (LGM, *n* = 6), and high-dose group (LGH, *n* = 6). In addition to the blank control group with the same volume of NS solution oral gavage, the other groups were given 7 mL/kg of 56-degree liquor twice a day for 10 days. Since the first day, the blank group and the model group were given distilled water by gavage at a dose of 10 mL/kg of body weight, positive control group was given BIFICO, a triplet living bacteria preparation (10^9^ CFU, according to the reported dose [25]), and low-dose group, middle-dose group, and high-dose group, respectively, received *Lindera aggregata* with the dosage of 1 g kg-1, 2 g/kg, and 3 g/kg, gavage administration, according to the reported dose [23, 26]. The treatments lasted for the entire experiment. 15 hours after the last oral alcohol gavage, blood samples were taken from portal vein and abdominal aorta to prepare serum, and liver samples were killed quickly.

### 2.3. Histology

At the end of the experiment, the rats (3 rats/group) were taken after blood collection and anesthetized with 3% pentobarbital sodium, the liver and the colon 3 cm away from the anus were removed, the left lobe of liver and colon were cleaned with PBS, the tissues were fixed with 4% formaldehyde and embedded with conventional paraffin, several slices (10 *μ*m thick) were made, and after HE staining, morphological observation was carried out under the light microscope to analyze the changes of histopathology.

For immunohistochemical staining, the obtained paraffin sections were subjected to rehydration treatment. Paraffin sections were then incubated with the primary antibody at 4°C overnight. Subsequently, biotinylated secondary antibodies were applied and treated together with an affinity-biotin-peroxidase complex, and DAB substrate was used to display the stained sections. The sections were then counterstained with hematoxylin. The stained sections were analyzed using light microscopy.

### 2.4. q-PCR

Total RNA was extracted from rat liver tissue by RNA Easy Fast Animal Tissue/cell Total RNA Extraction Kit (TIANGEN, Beijing, China), and RNA concentration was determined by NanoDrop ND-2000 (Thermo Fisher Scientific, Waltham, MA). Purified RNA was reverse transcribed into cDNA by PrimeScript™ RT reagent Kit with gDNA Eraser (TaKaRa, Dalian, China). RT-qPCR was performed using One-Step TB Green® PrimeScript™ RT-PCR Kit (TaKaRa, Dalian, China) and one-step method. Comparative CT method was used to calculate relative gene expression. The primers were synthesized by Sangon Biotech (Shanghai, China). Primers' information is supplemented in Table 1.

### 2.5. Western Blot

The frozen liver tissue is homogenized and used to extract the total protein of the tissue from the RIPA lysate (Beyotime, Haimen, China) containing protease inhibitor (Beyotime, Haimen, China). The nuclear protein was extracted by Nuclear and Cytoplasmic Protein Extraction Kit (Beyotime, Shanghai, China). The concentration of BCA protein was determined by BCA protein assay kit (Thermo Fisher Scientific, Waltham, MA). 40 *μ*g protein was added to 10% SDS-PAGE gel, separated by electrophoresis at a constant voltage of 80 V, and then transferred to PVDF membrane (MilliporeSigma, China). The membrane was blocked with 5% skim milk at room temperature for 2 hours. Anti-SIRT1 (Abcam, USA), anti-Nrf2 (Abcam, USA), anti-COX2 (Abcam, USA), anti-NF-*κ*B p65 (Abcam, USA), and anti-*β*-actin (Abcam, USA) were incubated overnight at 4°C. The next day after being washed 3 times with TBST buffer, PVDF membranes were treated with HRP-conjugated secondary antibodies (Huabio, Hangzhou, China) and incubated for 2 hours at room temperature. After washing with TBST buffer for 3 times, soak in developer, visualize in gel imaging system (Bio-Rad, Hercules, CA), and quantify with ImageJ software (1.50 d).

### 2.6. 16S rDNA Sequencing and Data Analysis

DNA was isolated from fecal samples of rats receiving different treatments using the E.Z.N.A.® Stool DNA Kit (D4015, Omega, Inc., USA) according to the manufacturer's instructions. Determination of concentration and purity was performed by ultraviolet spectroscopy. DNA integrity was also detected by agarose gel electrophoresis. The 16S rRNA V4 region was amplified using primers 515F (5′-GTGYCAGCMGCCGCGGTAA-3′) and 806R (5′-GGACTACHVGGGTWTCTAAT-3′). After purification and quantification of PCR products, sequencing libraries were generated by the Library Quantification Kit for Illumina (Kapa Biosciences, Woburn, MA, USA). PCR products were sequenced using the Illumina MiSeq platform (Emergency Department, People's Hospital of Tiantai County, Zhejiang, China).

fqtrim (v0.94) was used to filter the raw data and chimeric sequences were removed using the Vsearch algorithm to obtain clear sequences. Next, DADA2 was used to obtain the feature sequences. The samples were normalized according to SILVA (release 132) based on the Mothur algorithm and annotated with taxonomic information. The alpha diversity and beta diversity analyses of the normalized data were calculated by QIIME2. Principal coordinate analysis (PCoA), LEfSe analysis, and other analyses and visualizations were implemented by R software (v3.5.2). All experiments were performed in accordance with the reference [27–30] and manufacturer's instructions (LC-Bio Technologies, China).

### 2.7. Statistical Analysis

The data were analyzed with the software of IBM SPSS Statistics 25. The measurement data are expressed in mean ± standard ($\overset{¯}{x}\pm s$) deviation. Two-paired samples were tested by paired sample *T* test. Univariate analysis of variance was used for the comparison among groups. LSD test was used for homogeneity of variance and Dunnett T3 test was used for heterogeneity of variance. The data were visualized by Origin software; *p* < 0.05 was statistically significant.

## 3. Results

### 3.1. Ameliorative Effect of *Lindera aggregata* on Alcoholic Liver Injury

**Figure 1:**
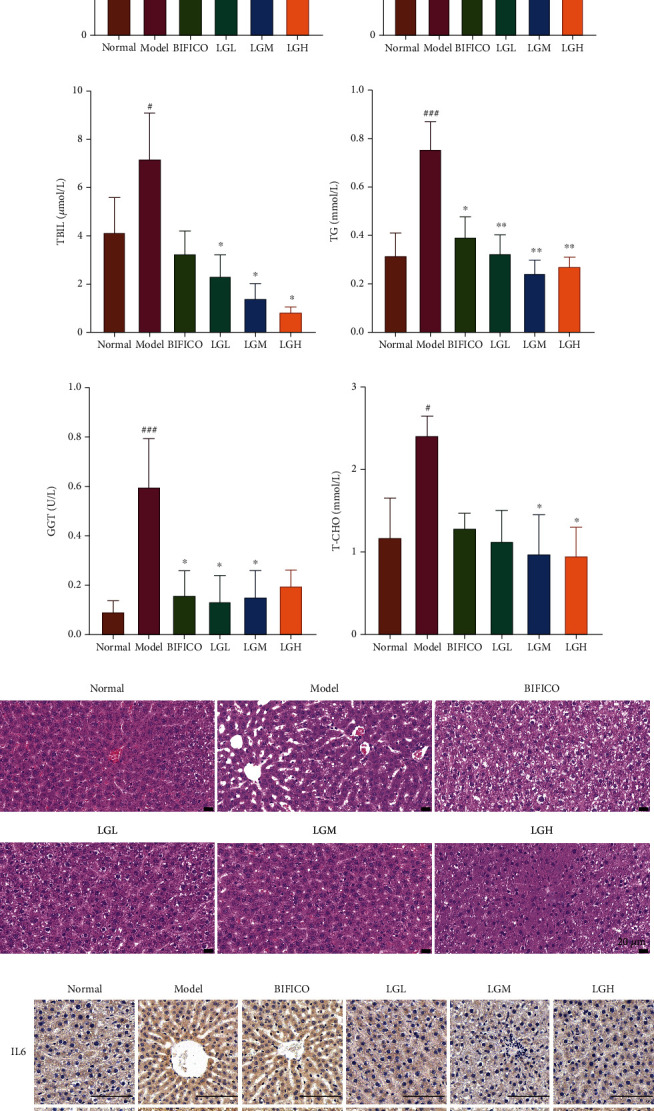
Effects of *Lindera aggregata* on serum and liver biochemical indexes in rats with alcoholic liver injury. (a) ALT: alanine aminotransferase; (b) AST: aspartate aminotransferase; (c) TBIL: total bilirubin; (d) TG: triglycerides; (e) GGT: gamma-glutamyl transferase; (f) T-CHO: total cholesterol. (g) Representative photomicrographs of hematoxylin- and eosin-stained sections of the liver (scale bar = 20 *μ*m). (h) Immunohistochemical staining of inflammatory factors IL6 and TNF-*α* in liver tissue (scale bar = 100 *μ*m). Data are shown as the mean ± SEM. Differences were assessed using ANOVA. ^#^*p* < 0.05, ^##^*p* < 0.01, and ^###^*p* < 0.001 compared with the normal group; ^∗^*p* < 0.05, ^∗∗^*p* < 0.01, and ^∗∗∗^*p* < 0.001 compared with the model group.

Serum AST, ALT, GGT, and TG levels were significantly increased in the model group (*p* < 0.001) (Figures 1(a), 1(b), 1(d), and 1(e)). Compared with the normal group, TBIL and T-CHO levels also increased (*p* < 0.05) (Figures 1(c) and 1(f)), which suggested the establishment of alcoholic liver model in rats. After treatment with Bifidobacterium triple preparation and alcohol extract of *Lindera aggregata*, all the indexes decreased to some extent. Serum TG concentration in the LGM group was significantly lower than the model group (*p* < 0.01), and the serum GGT concentration in the LGL group was also lower (*p* < 0.01). In terms of improving AST and ALT, *Lindera aggregata* appears to have the same effect as triple viable preparations. Compared with the model group, *Lindera aggregata* significantly reduced serum T-CHO and TBIL concentrations and showed dose-dependent (Figure 1).

### 3.2. Histological Analysis of Liver

HE staining of the liver demonstrated structural destruction of the hepatic lobules in the model, BIFICO, and LGL groups, as well as various size and spacing nodules of the hepatic lobules and surrounding desmoplasia. The LGM and LGH groups were similar to the normal group, and the structure of hepatic lobules was intact and clear. In the normal group, the hepatocytes were arranged radially with the central vein as the center. In the model group, the hepatic cords were disordered and inflammatory cells were infiltrating obviously. Compared with the model group, the BIFICO group and LGL group were improved, and the arrangement of hepatocytes in the LGM and LGH groups was almost normal (Figure 1(g)). In addition, we found that the rat liver in the model group had the highest positivity of IL6 and TNF-*α* by immunohistochemical staining of inflammatory factors IL6 and TNF-*α* in liver tissues from different groups, while the BIFICO group also had the same trend. And the treatment of *Lindera aggregata* led to the decrease of IL6 and TNF-*α* expression in liver tissues, which implied that *Lindera aggregata* could alleviate the liver damage caused by alcohol and inhibit the expression of inflammatory factors (Figure 1(h)).

### 3.3. *Lindera aggregata* Could Ameliorate Liver Injury Induced by Alcohol through SIRT1/Nrf2/NF-*κ*B/COX2 Signal Pathway

**Figure 2:**
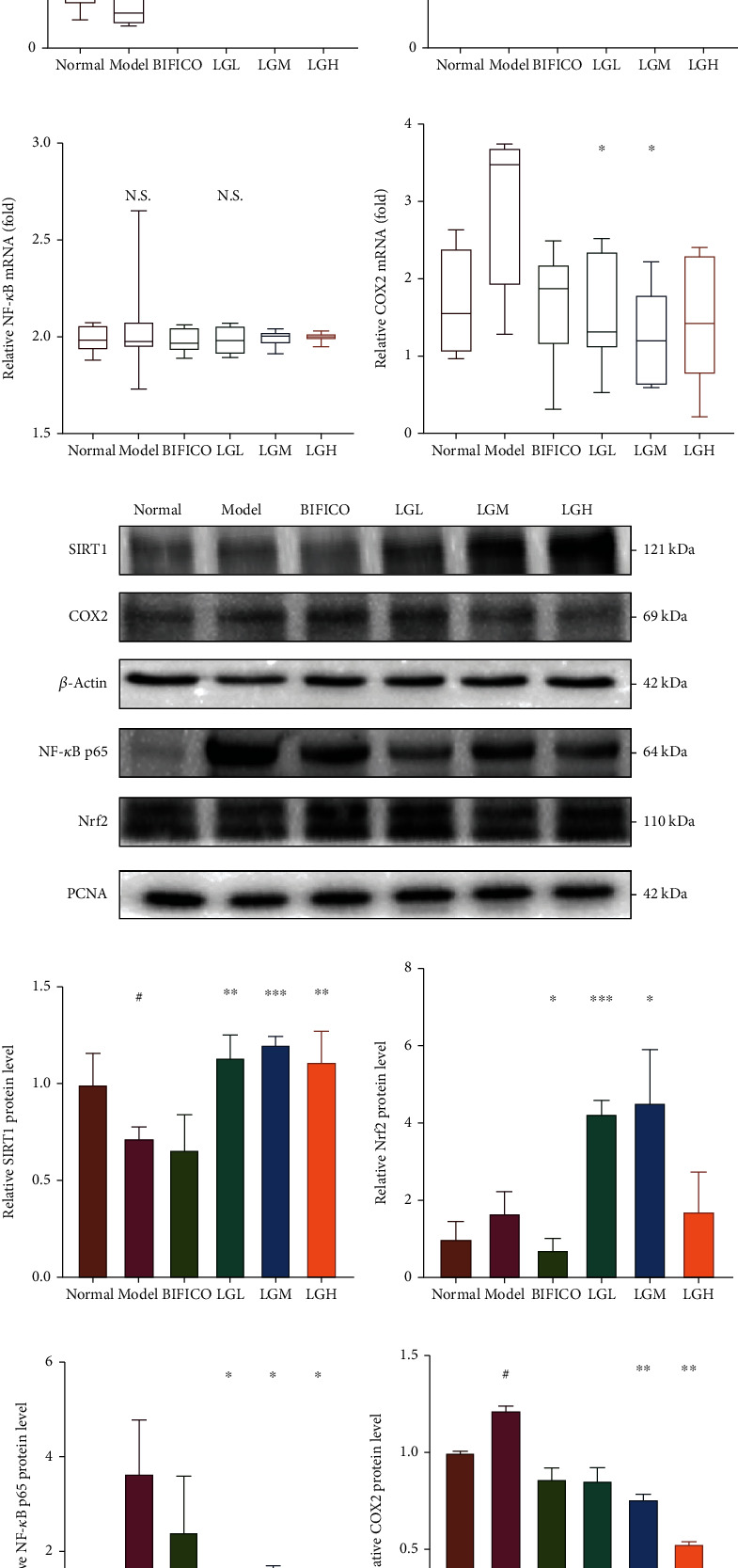
Effect of *Lindera aggregata* on the expression of inflammation-related and oxidative stress-related mRNA and protein in rats with alcoholic liver injury. (a) Relative mRNA expression of SIRT1 in rat liver. (b) Relative mRNA expression of Nrf2 in rat liver. (c) Relative mRNA expression of NF-*κ*B in rat liver. (d) Relative mRNA expression of COX2 in rat liver. (e) Western blotting was used to detect the protein levels of key molecules in the oxidative stress and inflammatory signaling pathways (SIRT1, Nrf2, NF-*κ*B, and COX2) in the liver among the different groups. (f) SIRT1 protein level in rat liver. (g) Nrf2 protein level in rat liver. (h) NF-*κ*B protein level in rat liver. (i) COX2 protein level in rat liver. Data are shown as the mean ± SEM. Statistical analysis of intergroup data was performed using Tukey's test or the Mann–Whitney test. ^#^*p* < 0.05, ^##^*p* < 0.01, and ^###^*p* < 0.001 compared with the normal group; ^∗^*p* < 0.05, ^∗∗^*p* < 0.01, and ^∗∗∗^*p* < 0.001 compared with the model group.

In order to elucidate the molecular mechanism of *Lindera aggregata*, we first analyzed the mRNA expression of NF-*κ*B, SIRT1, Nrf2, and COX2 in rat liver by q-PCR (Figures 2(a)–2(d)). The results showed that the expression of SIRT1, which was inhibited by alcohol, was upregulated by the intervention of different concentrations of *Lindera aggregata* (Figure 2(a)), and the expression of Nrf2 was also upregulated by high dose of *Lindera aggregata* (Figure 2(b)). Additionally, alcohol induced the expression of COX2 in the liver of rats, which was reversed by the treatment of *Lindera aggregata* (Figure 2(d)).

Western blotting showed a similar trend (Figure 2(e)), with the increased expression of SIRT1 and Nrf2 stimulated by alcohol, which implied the effect of *Lindera aggregata* on resisting oxidative stress in vivo (Figures 2(f) and 2(g)). We found that the treatment of *Lindera aggregata* could significantly downregulate the DNA binding activity of NF-*κ*B and decrease the alcohol-induced nuclear translocation of NF-*κ*B (Figure 2(h)). At the same time, the expression of COX2 (Figure 2(i)), which is related to inflammation, was also downregulated by the intervention of *Lindera aggregata*. These data suggest that ethanol extracts of *Lindera aggregata* may resist the effects of alcohol by activating the expression of antioxidant and anti-inflammatory molecules.

### 3.4. Effect of *Lindera aggregata* Treatment on Intestinal Community Composition in Mice

**Figure 3:**
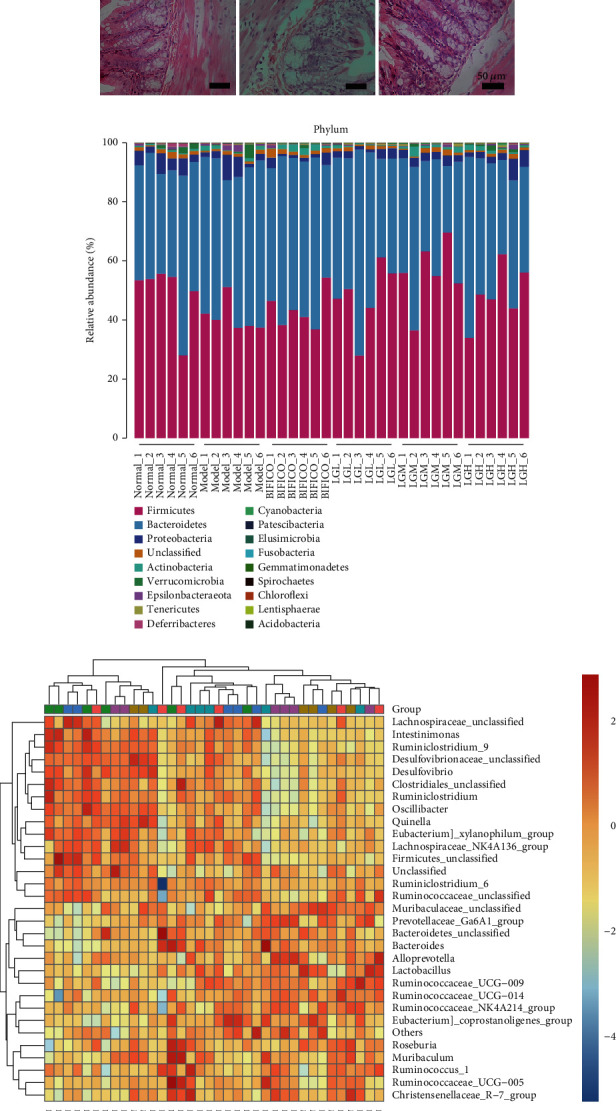
Significant changes of gut microbiota at phyla level and genus level in rats. (a) Representative photomicrographs of hematoxylin- and eosin-stained sections of the gut (scale bar = 50 *μ*m). (b) The composition of gut microbiota of rats in different treatment groups at the phylum level. (c) The relative abundance of gut microbiota of rats in different treatment groups at the genus level.

HE staining of the intestine showed no obvious pathological changes in each group (Figure 3(a)). We suspect that although frequent alcohol stimulation does not cause significant pathological damage to the gut, the microbiome is inevitably affected. NovaSeq platform was used to analyze the V4 region of bacterial small subunit (16S) rRNA gene commonly used in intestinal microbiota research and to study the effect of *Lindera aggregata* on intestinal microflora. After filtering out the unqualified sequence, through QIIME 2 analysis, we use DADA2 to denoise the amplified subdata, cluster with 100% similarity, remove and correct the low-quality sequence, and obtain the amplicon sequence variants and then deredundancy, resulting in a total of 1794004 features from 36 rat stool samples for further analysis.

**Figure 4:**
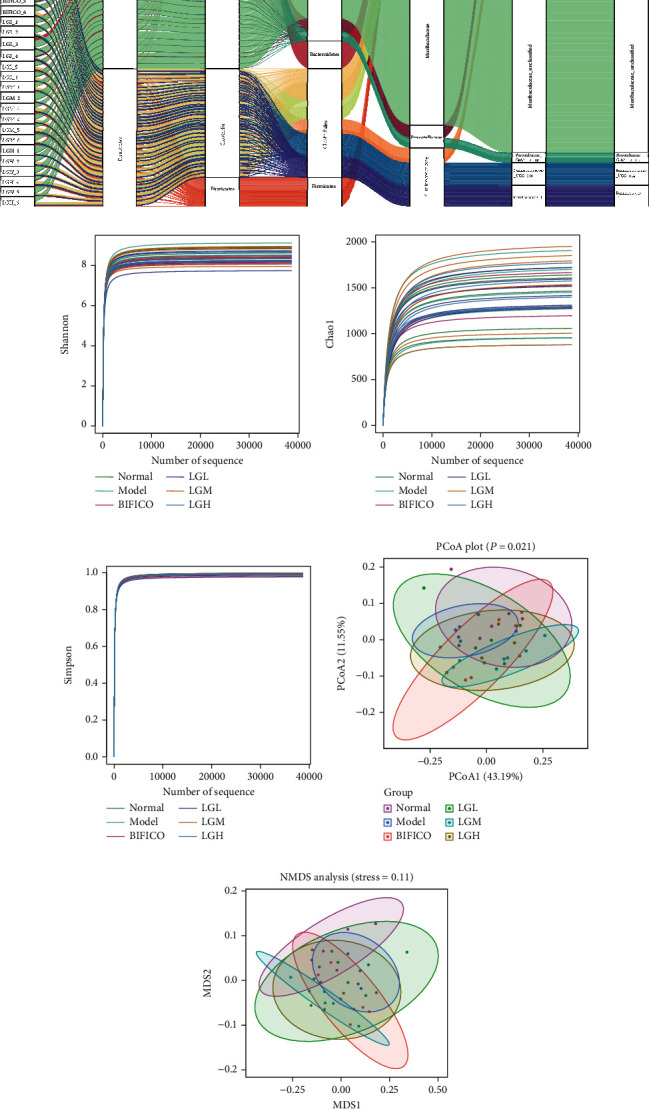
Regulation of *Lindera aggregata* on the diversity of gut microbiota in rats. (a) Sankey diagram shows the abundance and affiliation of microorganisms at different taxonomic levels in the gut of rats under different treatment strategies. (b) Shannon rarefaction curves. (c) Chao1 rarefaction curves. (d) Simpson rarefaction curves. (e) Principle coordinate analysis (PCoA) analysis based on weighted UniFrac distance of gut microbiota of rats in different treatment groups. ANOSIM was used for similarities in PCoA. (f) Nonmetric multidimensional scaling (NMDS) analysis based on weighted UniFrac distance of gut microbiota of rats in different treatment groups. When stress is less than 0.05, the data is considered well representative.

We analyze the bacterial flora of six groups of samples at the phyla level and identify a total of 18 phyla.  Figure 3(b) shows their relative abundance in each sample. Compared with the normal group, alcohol intake significantly decreased *Firmicutes* and *Proteobacteria* in the intestine and significantly increased *Bacteroidetes*. At the same time, we also isolated 347 bacterial genera, and according to the genus level, we drew the distribution heatmap of top 30 in each sample (Figure 3(c)). *Muribaculaceae_unclassified* and *Lachnospiraceae_NK4A136_group* were the two abundant genera. Alcohol caused a significant decrease in the relative bacterial populations of *Muribaculaceae_unclassified*, *Firmicutes_unclassified*, and *Ruminiclostridium_9*, but a significant increase in the abundance of *Muribaculaceae_unclassified*, *Lactobacillus*, and *Ruminococcaceae_UCG-014.* After *Lindera aggregata*, the change was reversed and the effect was better than that of the conventional bacteria-disordered drug preparation, the Bifidobacterium triplet preparation. The changes between these groups suggested that Lindera aggregation treatment could regulate the structure of the intestinal microflora of alcoholic liver disease to a normal level (Figure 4(a) and Supplementary Figure 1 and 2).

### 3.5. Alpha Diversity Analysis

When the dilution curve flattens out, the sequence depth is considered to be sufficient to cover all species in the sample for further data analysis. Alcohol administration inhibited microbial abundance and diversity (Chao1) in the gut compared with healthy control rats. Comparing the diversity index of each group, we found that there was a change in the bacterial diversity between the normal group and the model group, but the BIFICO treatment could not improve the bacterial diversity of the intestinal tract. After treatment with different dosages of *Lindera aggregata*, the richness and evenness reflected by Chao1 index, Shannon index, and Simpson index increased significantly (Figures 4(b)–4(d)). These results suggest that ethanol extract of *Lindera aggregata* can improve the microbial diversity in rats with alcohol-induced liver injury.

### 3.6. *β*-Diversity Analysis

The *β*-diversity index also showed that the samples of the normal group and BIFICO group and different dosage of *Lindera aggregata* had strong differentiation, with statistical significance (*p* < 0.05). The results of weighted principal coordinate analysis (PCoA) and unweighted UniFrac distance showed that there were only a few overlapping regions between the normal group and model group, indicating a higher number of OTUs with difference (Figure 4(e)). The weighted PCoA showed that the structure of intestinal flora responded to the obvious changes in the treatment of *Lindera aggregata* (Figure 4(e)). The correlation difference mainly appeared on PCoA1, which accounted for the largest proportion of the total variation (43.19%).

Weighted PCoA was also used to distinguish the microbial composition of the gut under different treatment modes, and the high-dose *Lindera aggregata* treatment group and BIFICO treatment group showed a mostly overlapping and similar trend. In addition, the low-, middle-, and high-dose groups had the same tendency as the normal group on different analysis levels, and we found that there was a partial overlap between the triple viable preparation of the BIFICO treatment group and the normal group; it is suggested that *Lindera aggregata* might have the effect of regulating the intestinal flora homeostasis and promoting the proportion of the flora to be normal. Nonmetric multidimensional scale analysis (NMDS) showed that both the model group and control group had obvious isolated clusters outside the overlap region, which indicated that there was a certain change tendency of microbial composition structure induced by alcohol. The bacterial community structure of different doses of *Lindera aggregata* treatment group was significantly more inclined to the normal group and the BIFICO group (Figure 4(f)).

### 3.7. Difference of Microflora under Different Treatments

LEfSe is an analytical tool for the discovery and interpretation of biomarkers for high-dimensional data. The method synthesizes the statistical difference analysis and the influence score of the different species on the grouping result and emphasizes the statistical significance and biological correlation. The threshold of LEfSe analysis was set to LDA index > 3, *p* < 0.05. Compared with normal rats, analyze the changes in the composition of the intestinal microbiota of rats in the model group, the common live bacteria preparation group, and the different doses of *Lindera aggregata* treatment group to determine the bacteria genera that are enriched in the two groups. The relative abundance of *Bifidobacterium*, *Bifidobacteriaceae*, *Bifidobacteriales*, *Bifidobacterium_pseudolongum*, and *Coprococcus_2* increased after ethanol intervention (Supplementary Figure 3 and 4).

**Figure 5:**
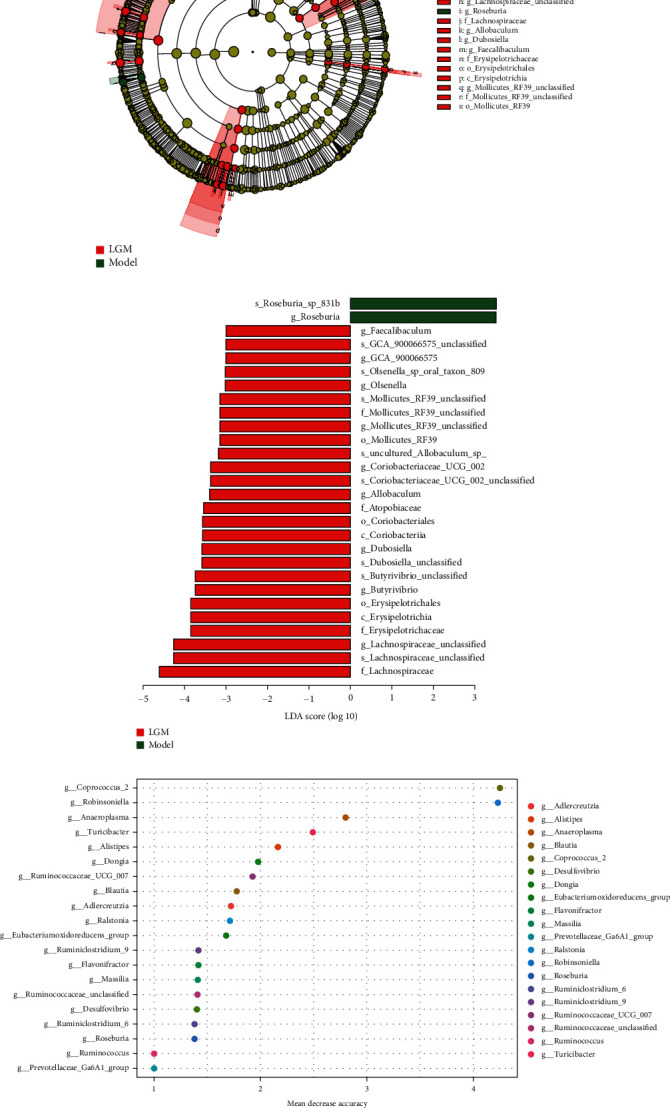
The core bacteria with different microbiota were analyzed by LEfSe. (a) Cladogram showed the rich taxa in gut microbiota between the model group and LGM group. (b) LDA score of differential microbial taxon between the model group and LGM group. (c) Random forest analysis of important microbial units between the LGM group and model group.

After treatment with BIFICO, the dominant groups of bacteria did not change significantly. However, the relative abundance of *Ruminococcus_1*, *Erysipelotrichaceae*, *Erysipelotrichia*, and *Erysipelotrichales* increased in the low-dose group and *Ruminococcus_2*, *Ruminococcaceae*, *GCA_900066575*, and *Mollicutes_RF39* increased in the high-dose group compared with the model group (Supplementary Figure 5-10). We found that after the treatment of alcoholic liver rats with moderate dosage of *Lindera* root, the content of intestinal flora changed complicatedly, the abundance of a large number of flora increased, and the abundance of predominant flora was reversed under earlier stimulation. *Lachnospiraceae*, *Erysipelotrichaceae*, *Erysipelotrichia*, *Butyrivibrio*, *Dubosiella*, *Coriobacteriia*, *Atopobiaceae*, and other bacteria species had the most obvious changes in abundance, and LDA values are greater than 4 (Figures 5(a) and 5(b)). At the same time, the identification of different bacteria genera between the two samples based on random forest method also confirmed this point (Figure 5(c) and Supplementary Figure 11-14).

In order to further explore the relationship between these core influence groups, we analyzed the abundance and variation of different bacteria in different samples by SPARCC method and calculated the microbial abundance of top 30 in bacterial genus level, the correlation between the two dominant bacteria groups was obtained, and then the heatmap was drawn by the correlation (Supplementary Figure 15). By using redundancy analysis (RDA), we identified the key variables that responded to the different processing (Supplementary Figure 16 and 17).

### 3.8. Predictive Function Analysis

**Figure 6:**
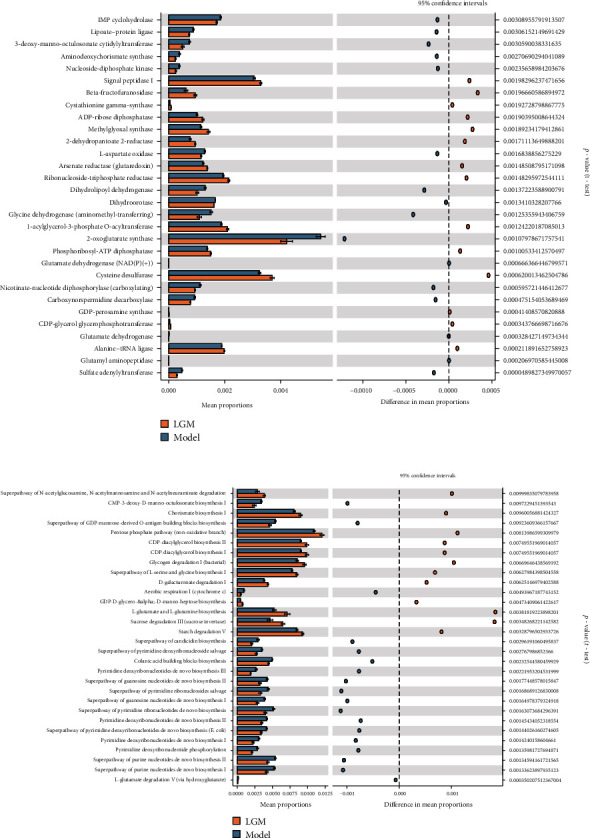
The function of microbial genes predicted by PICRUSt2 based on annotations from EC and KEGG databases. (a) PICRUSt2 analysis in the annotation of EC database. Functional predictions for the gut microbiome of the LGM group and model group. (b) PICRUSt2 analysis in the KEGG pathways. Functional predictions for the gut microbiome of the LGM group and model group.

To further investigate the influence of gut microbes under different treatment strategies and the potential functions possessed by these differential microflora, and to determine the interpretation of these metabolic pathways on the association between dysbiosis and alcoholic liver. We used PICRUSt2 to predict the possible function of bacterial population genes and annotated the bacterial population gene function by COG, EC, and KEGG databases. We then used STATistical Analysis of Metagenomic Profiles (STAMP) for difference analysis with the aim of identifying significant differences in gene function between groups. We found that the therapeutic dose of ethanol extract of *Lindera aggregata* also had a key effect on the improvement and balance of intestinal microflora, and the effect was not dose-dependent. In comparison with the model group, the moderate-dose group had the most significant improvement in intestinal flora abundance and gene function. We found a total of 100 different KEGG pathways, and the enrichment of pentose phosphate pathway, L-glutamate and L-glutamine biosynthesis, CDP diacylglycerol biosynthesis, and other pathways increased in the treatment group (Figure 6(a)). In the high-dose group and the low-dose group, 13 and 9 pathways were predicted, respectively, with only a slight change in the enrichment trend (Supplementary Figure 18 and 19).

**Figure 7:**
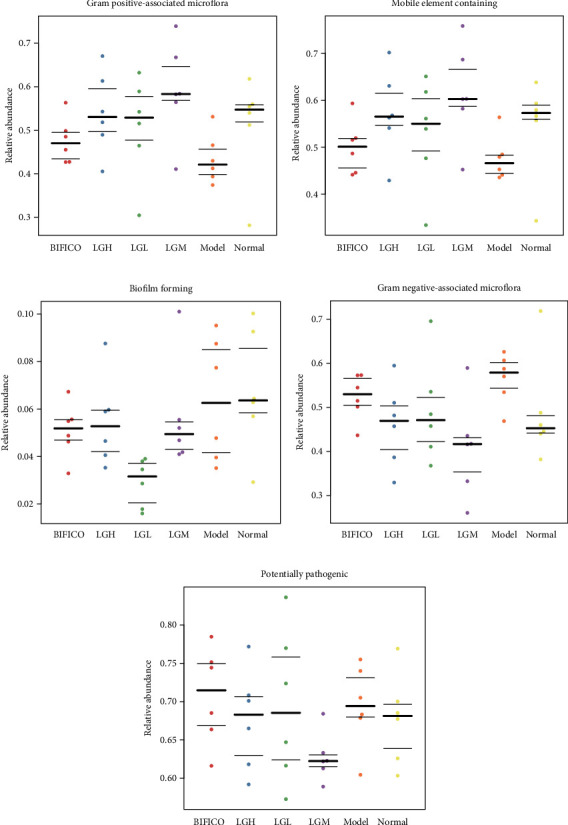
Phenotypic prediction of microflora. (a) Relative expression abundance of Gram-positive-associated microflora. (b) Relative expression abundance of microflora associated with mobile element containing. (c) Relative expression abundance of microflora associated with biofilm forming (d) Relative expression abundance of Gram-negative-associated microflora. (e) Relative expression abundance of microflora associated with potential pathogenesis.

At the same time, based on the annotation of COG and EC databases, after treatment with moderate dose of *Lindera aggregata* in rats with alcoholic liver injury, we found that the enrichment of metabolism-related functions such as signal peptidase I, beta-fructofuranosidase, cysteine desulfurase, and pimeloyl-ACP methyl ester carboxylesterase was significantly increased, while the enrichment of biosynthesis-related functions such as 2-oxoglutarate synthase, nicotinate-nucleotide diphosphorylase (carboxylating), and Fe^2+^ or Zn^2+^ uptake regulation protein was significantly decreased (*p* < 0.01) (Figure 6(b) and Supplementary Figure 20-24). Next, we used BugBase to classify the microbial community according to seven phenotypes and to predict the phenotypes of the flora. Finally, we found that after treatment with *Lindera aggregata* and probiotics, the abundance of Gram-positive-associated microflora and microflora associated with mobile element contained increased significantly, while the abundance of microflora associated with biofilm formation and Gram-negative-associated microflora decreased. At the same time, only moderate doses of *Lindera aggregata* treatment reduced the relative abundance of microflora associated with potentially pathogenic under all treatment strategies (Figures 7(a)–7(e)).

Furthermore, these evidences suggest that appropriate dosage is also a key factor to consider in the treatment of alcoholic liver disease with Chinese herbal extracts based on the effect of the liver-gut axis effect.

## 4. Discussion

In this study, we constructed a rat model of alcohol-induced liver injury to evaluate the improvement effect of ethanolic extract of *Lindera aggregata* at different doses on liver function from the levels of oxidative stress and inflammation. As rich in alkaloids, volatile oil, flavonoids, sesquiterpene esters, and other chemicals of natural products, *Lindera aggregata* can reduce the level of alcohol-induced dyslipidemia, improve triglyceride, cholesterol, ALT, AST, GGT, and TBIL in rats with alcoholic liver injury, reduce liver oxidative stress and proinflammatory factor expression, balance the gut microbiota, and drive beneficial bacteria to occupy the dominant ecological niche. In addition, the relative abundance of *Muribaculaceae*, *Lachnospiraceae_NK4A136*, *Firmicutes*, *Lachnospiraceae*, *Lactobacillus*, *Actinobacteria*, *Spirochaetes*, and *Elusimicrobia* genera was positively correlated with the healthy liver status of rats, while the relative abundance of *Bacteroidetes*, *Ruminococcaceae_UCG-014*, *Ruminococcus_1*, *Epsilonbacteraeota*, *Tenericutes*, and *Cyanobacteria* genera was negatively correlated with the healthy liver status of rats. The results suggest that *Lindera aggregata* may be a potential therapeutic adjuvant for the treatment of alcohol-induced liver injury and intestinal dysfunction.

SIRT1 has been shown to protect hepatocytes from alcohol-induced liver damage by activating several signaling pathways that regulate energy metabolism and stress responses [31]. In this study, compared with the model group, the treatment of *Lindera aggregata* significantly reversed the inhibition of SIRT1 and Nrf2 expression induced by alcohol stimulation and increased the nuclear translocation of Nrf2. At the same time, we found that *Lindera aggregata* could negatively regulate the DNA binding activity of NF-*κ*B and decrease the content of NF-*κ*B p65 transferred into the nucleus. COX2, which is related to inflammation, was also negatively regulated by the intervention of *Lindera aggregata* intervention. These results suggest that compounds in *Lindera aggregata* may further improve liver injury by activating the expression of SIRT1 and Nrf2, regulating oxidative stress and inflammation in the alcohol-induced liver. SIRT1 was found to protect the liver from drug-induced liver injury and could also improve the lipopolysaccharide-induced liver injury by activation of NF-*κ*B [32]. SIRT1-related cascade reaction may act as upstream of Nrf2 signaling pathway, increase Nrf2 transcription and activation, promote Nrf2 transposition into cell nucleus, and activate antioxidant gene expression [33]. In some of the studies using herbs to treat oxidative stress-induced liver injury, Luo et al. found that luteolin directly reduced NF-*κ*B activation by activating SIRT1 to promote NF-*κ*B deacetylation. Furthermore, SIRT1 can reduce ROS production and accumulation by activating Nrf2-related pathways [34]. This further demonstrates the feasibility of using herbal extracts to treat alcoholic liver injury with high levels of inflammation and oxidative stress. ROS induced by oxidative stress can regulate inflammatory cytokines through NF-*κ*B signaling pathway, while activated NF-*κ*B regulates the transcription and translation of many genes, such as inflammatory and chemokine, and upregulates the expression of COX2, leading to inflammation [35].

Gut-liver interactions have been proven to be critical in the pathogenesis of alcohol-mediated liver disease. The stability of gut-liver axis regulation system is related to the integrity of intestinal barrier function, the normal function of liver, intestinal immune response, and systemic inflammation. In our study, we found that the relative abundance of *Muribaculaceae*, *Liposomae_NK4A136*, *Fireplaces*, *Liposomae*, *Lactobacillus*, *Actinobacteria*, *Spirochaetes*, and *Elusimicrobia* significantly decreased and the populations of *Bacteroidetes*, *Ruminococcaceae_UCG-014*, *Ruminococcus_1*, *Epsilonbacteraeota*, *Tenericutes*, and *Cyanobacteria* significantly increased in rats stimulated by alcohol alone. Similar to most studies, *Firmicutes* and *Bacteroidetes* account for more than 90% of the relative abundance of intestinal flora, but alcohol can also induce significant changes in flora. In line with previous studies on the molecular mechanisms of liver injury, high-dose *Lindera aggregata* treatment did not significantly modulate the intestinal flora in rats, suggesting that the treatment of traditional Chinese herbs requires an appropriate dose; high therapeutic doses may irritate the intestinal environment, which is not conducive to the growth of beneficial bacteria. The medium dose of *Lindera aggregata* had the best effect on the improvement of blood indexes of liver and the growth of beneficial bacteria, and the Shannon index and Chao1 index were increased; the higher *α*-diversity combined with LEfSe analysis showed that beneficial bacteria gradually occupied the dominant niche and through the secondary metabolites to improve the intestinal flora disorder.

Our results show that elevated levels of the inflammatory cytokines COX2 and NF-*κ*B were associated with an increased relative abundance of disease-associated bacteria in rats; the low expression of the antioxidant factor SIRT1 and the anti-inflammatory-related factor Nrf2 was similarly associated with a decrease in the relative abundance of bacteria colonized in a healthy gut. Moderate dose of *Lindera aggregata* significantly increased the level of SIRT1 and Nrf2, decreased the expression of COX2 and NF-*κ*B, improved the inflammatory status and oxidative stress, and accompanied with the increase of relative abundance of *Lactobacillus*, *Muriculaceae*, *Firmicutes*, *Lachnospiraceae*, and *Ruminococcaceae*. Changes in the abundance, function, and dominant niche of circulating microorganisms in the body were also associated with impaired immune responses in patients with alcoholic liver injury, according to a comparative analysis of the gut microbiota in patients with alcoholic hepatitis [36]. For most patients with liver disease deterioration, the intestinal microflora is characterized by an increase in the relative abundance of *Enterobacteriaceae* and a decrease in the relative abundance of *Lachnospiraceae* and *Ruminococcaceae*, accompanied by serious intestinal microecological imbalance and increased endotoxin content in blood. Leclercq et al. studied the potential reversibility of alcohol-induced microecological disorders in patients with simple alcohol intake (no obvious pathological condition) and found that about 40% of the patients had intestinal microflora changes, which was characterized by a decrease in the abundance of *Ruminococcaceae*, high intestinal permeability. The abundance of *Ruminococcaceae* increased when alcohol intake was stopped [37].

With the stimulation of alcohol, the relative abundance of bacteria associated with Gram-positive bacteria and mobile element containing decreased significantly in the gut, while the relative abundance of bacteria associated with Gram-negative bacteria and biofilm formation increased significantly; these changes can be reversed by probiotics and treatment with different doses of *Lindera aggregata*. Only in the medium dose of *Lindera aggregata* intervention, the increased pathogenic bacteria content was significantly reduced. The bacteriological biofilm is a kind of life phenomenon that bacteria adapt to the natural environment for survival. It has natural resistance to antibiotics and immunity. Disease factors and antibiotic resistance genes from mobile genetic elements can be transferred to and from surrounding bacteria for interchange, and foreign genes obtained through this mechanism can increase adaptability by acquiring new functions [38]. Long-term alcohol stimulation leads to excessive growth of Gram-negative bacteria in the gut, which increases production and release of endotoxin [39]. Therefore, the treatment of intestinal dysbacteriosis and pathogenic bacterial translocation may help prevent the development of ALI [40]. Similarly, treatment with *Lindera aggregata* extract reduces the relative abundance of potential pathogens and inflammation levels, and the reoccupation of dominant niches by beneficial bacteria may also be mediated by the same mechanism.

Our prediction of gut microbiota function in rats showed that the treatment of *Lindera aggregata* promoted the biosynthesis and metabolic pathway-related microbial functions. This means that the herb can improve the balance of the gut microbiota and maintain the integrity of the intestinal barrier by adjusting the proportion of microorganisms and their metabolism, thus reducing the damage caused by alcohol. In response to serum biochemical analysis, different treatment and treatment strategies led to changes in the predominant flora, as well as changes in the microbial metabolism. Frequent alcohol consumption can reduce levels of microbial metabolism products such as SCFAs and sulfides, as well as antioxidant fatty acids [41]. Serum levels of bile acid and benzoic acid metabolites associated with secondary microbial metabolism also changed [42]. Microbial metabolism products also affect peripheral inflammation.

We demonstrated that *Lindera aggregata* ethanol extracts could alleviate oxidative stress and inflammation through the SIRT1/Nrf2/NF-*κ*B pathway. We found that frequent alcohol stimulation may contribute to the reduction of beneficial bacteria and increase in the number of harmful bacteria in the gut. Similarly, we have demonstrated that *Lindera aggregata* may modulate the intestinal flora disturbance caused by alcohol intake and that appropriate doses of Chinese herbal medicine may be effective.

## 5. Conclusion

In conclusion, the present study demonstrated the therapeutic efficacy of the traditional Chinese herbal medicine, *Lindera aggregata,* based on the effects of its alcoholic extract on the liver-gut axis for alcohol-induced liver injury and intestinal microecological disorders using animal interventions and microbiomic analysis. Our study provides reliable theoretical and practical evidence for *Lindera aggregata* as an effective therapeutic strategy for alcoholic liver injury with intestinal system disorders in the future. In addition to demonstrating the biological function of *Lindera aggregata*, this study will require significant work in the future to identify and elaborate the ratio of active components. The improvement of the liver and gut could not be well demonstrated for the apparent crosstalk between the two. More long-term interventions are needed to further illustrate the complex interactions in the gut-liver axis. We expect that in the future, we can further demonstrate the effective probiotic value of *Lindera aggregata* on the liver and intestine in terms of compounds, miRNAs, and plant-derived nanoparticles.

## Figures and Tables

**Table 1:** Primers sequences used in the study.

Gene names	Forward primer 5′–3′	Reverse primer 5′–3′
GAPDH	GGTGAAGGTCGGTGTGAACG	CTCGCTCCTGGAAGATGGTG
COX2	AGGTCATCGGTGGAGAGGTGTA	GCGGATGCCAGTGATAGAGTGT
SIRT1	AACCTCTGCCTCATCTACA	GCTCTCAACATTCCTATAAGTC
Nrf2	GACAGAGATGGACAGCAAT	GACTAATGGCAGCAGAGG
NF-*κ*B	ATCCAACACAGGCATCAC	CCAGCAGCATCTTCACAT

## Data Availability

The sequence data are available in the Sequence Read Archive (SRA) under accession number SRP341289.

## Supplementary Materials

Supplementary Materials Supplementary Figure 1: genus phylotree of the intestinal flora of each group of rats. Supplementary Figure 2: interaction of the intestinal flora at the phylum level of Circos. Supplementary Figures 3 and 4 model group vs. normal group for differential species analysis. Supplementary Figures 5 and 6: BIFICO group vs. model group for differential species analysis. Supplementary Figures 7 and 8: LGL group vs. model group for differential species analysis. Supplementary Figures 9 and 10: LGH group vs. model group for differential species analysis. Supplementary Figure 11: based on random forest algorithm, the normal group was compared with the model group for feature species analysis. Supplementary Figure 12: based on random forest algorithm, the BIFICO group was compared with the model group for feature species analysis. Supplementary Figure 13: based on random forest algorithm, the LGL group was compared with the model group for feature species analysis. Supplementary Figure 14: based on random forest algorithm, the LGH group was compared with model group for feature species analysis. Supplementary Figure 15: correlation analysis of differential species. Supplementary Figure 16: redundancy analysis of flora at the phylum level. Supplementary Figure 17: redundancy analysis of flora at the genus level. Supplementary Figure 18: prediction of pathways for differential species function in the LGL group vs. model group. Supplementary Figure 19: prediction of pathways for differential species function in the LGH group vs. model group. Supplementary Figure 20: prediction of pathways based on annotation of the COG database on the function of differential species in the LGM group vs. model group. Supplementary Figure 21: prediction of pathways based on annotation of the EC database on the function of differential species in the LGL group vs. model group. Supplementary Figure 22: prediction of pathways based on annotation of the COG database on the function of differential species in the LGL group vs. model group. Supplementary Figure 23: prediction of pathways based on annotation of the COG database on the function of differential species in the LGH group vs. model group. Supplementary Figure 24: prediction of pathways based on annotation of the EC database on the function of differential species in the LGH group vs. model group.
