# Supplementary figures and images for: The Ethanolic Extract of Lindera aggregata Modulates Gut Microbiota Dysbiosis and Alleviates Ethanol-Induced Acute Liver Inflammation and Oxidative Stress SIRT1/Nrf2/NF-κB Pathway
Source: Oxid Med Cell Longev. 2022 Dec 20;2022:6256450. doi: 10.1155/2022/6256450 (PMC9794438; doi:10.1155/2022/6256450)

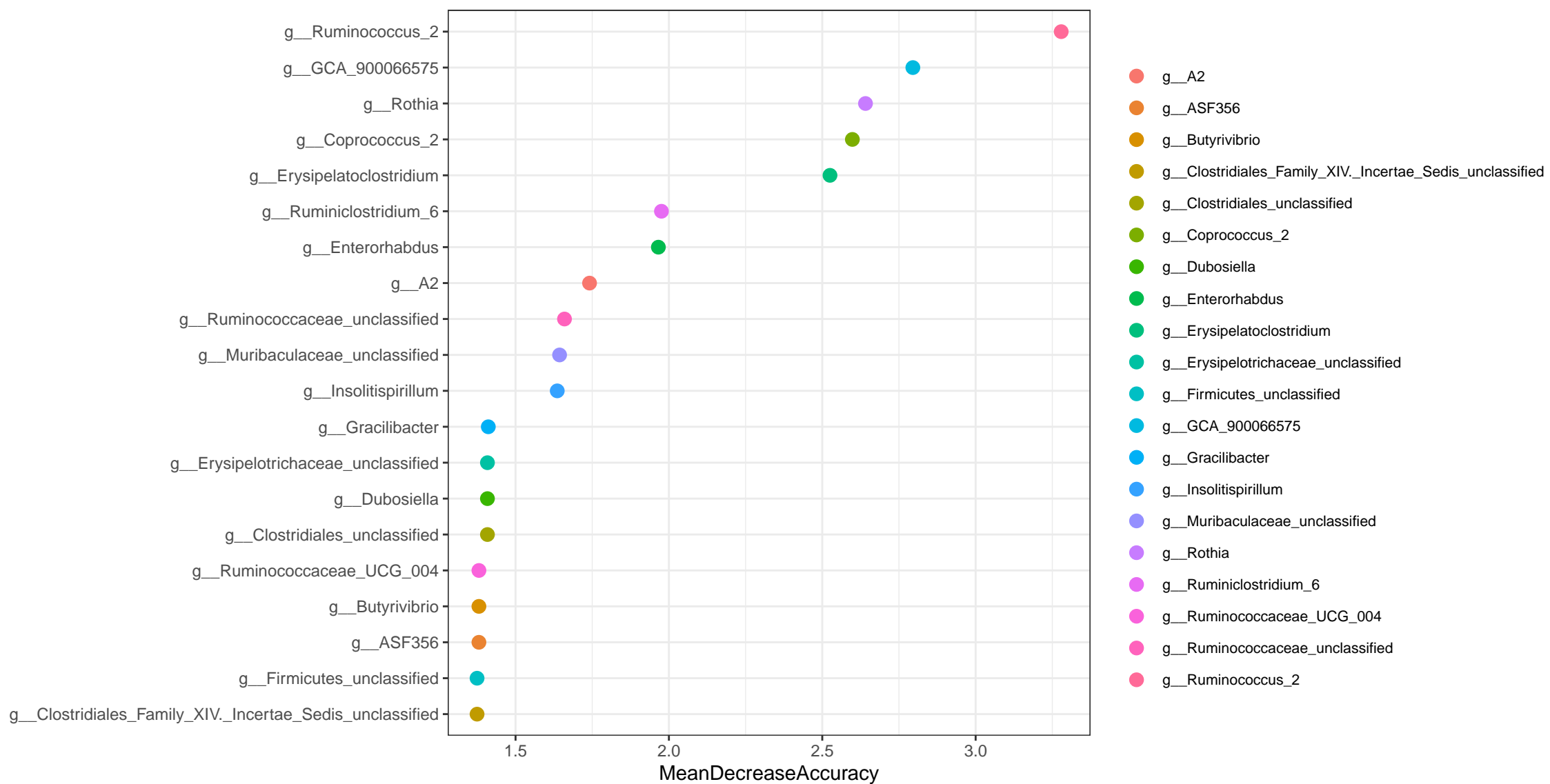

Supplement: Supplementary Materials — Supplementary Figure 1: genus phylotree of the intestinal flora of each group of rats. Supplementary Figure 2: interaction of the intestinal flora at the phylum level of Circos. Supplementary Figures 3 and 4 model group vs. normal group for differential species analysis. Supplementary Figures 5 and 6: BIFICO group vs. model group for differential species analysis. Supplementary Figures 7 and 8: LGL group vs. model group for differential species analysis. Supplementary Figures 9 and 10: LGH group vs. model group for differential species analysis. Supplementary Figure 11: based on random forest algorithm, the normal group was compared with the model group for feature species analysis. Supplementary Figure 12: based on random forest algorithm, the BIFICO group was compared with the model group for feature species analysis. Supplementary Figure 13: based on random forest algorithm, the LGL group was compared with the model group for feature species analysis. Supplementary Figure 14: based on random forest algorithm, the LGH group was compared with model group for feature species analysis. Supplementary Figure 15: correlation analysis of differential species. Supplementary Figure 16: redundancy analysis of flora at the phylum level. Supplementary Figure 17: redundancy analysis of flora at the genus level. Supplementary Figure 18: prediction of pathways for differential species function in the LGL group vs. model group. Supplementary Figure 19: prediction of pathways for differential species function in the LGH group vs. model group. Supplementary Figure 20: prediction of pathways based on annotation of the COG database on the function of differential species in the LGM group vs. model group. Supplementary Figure 21: prediction of pathways based on annotation of the EC database on the function of differential species in the LGL group vs. model group. Supplementary Figure 22: prediction of pathways based on annotation of the COG database on the function of differential spe [file 6256450.f1.zip › 6256450.f10.pdf]

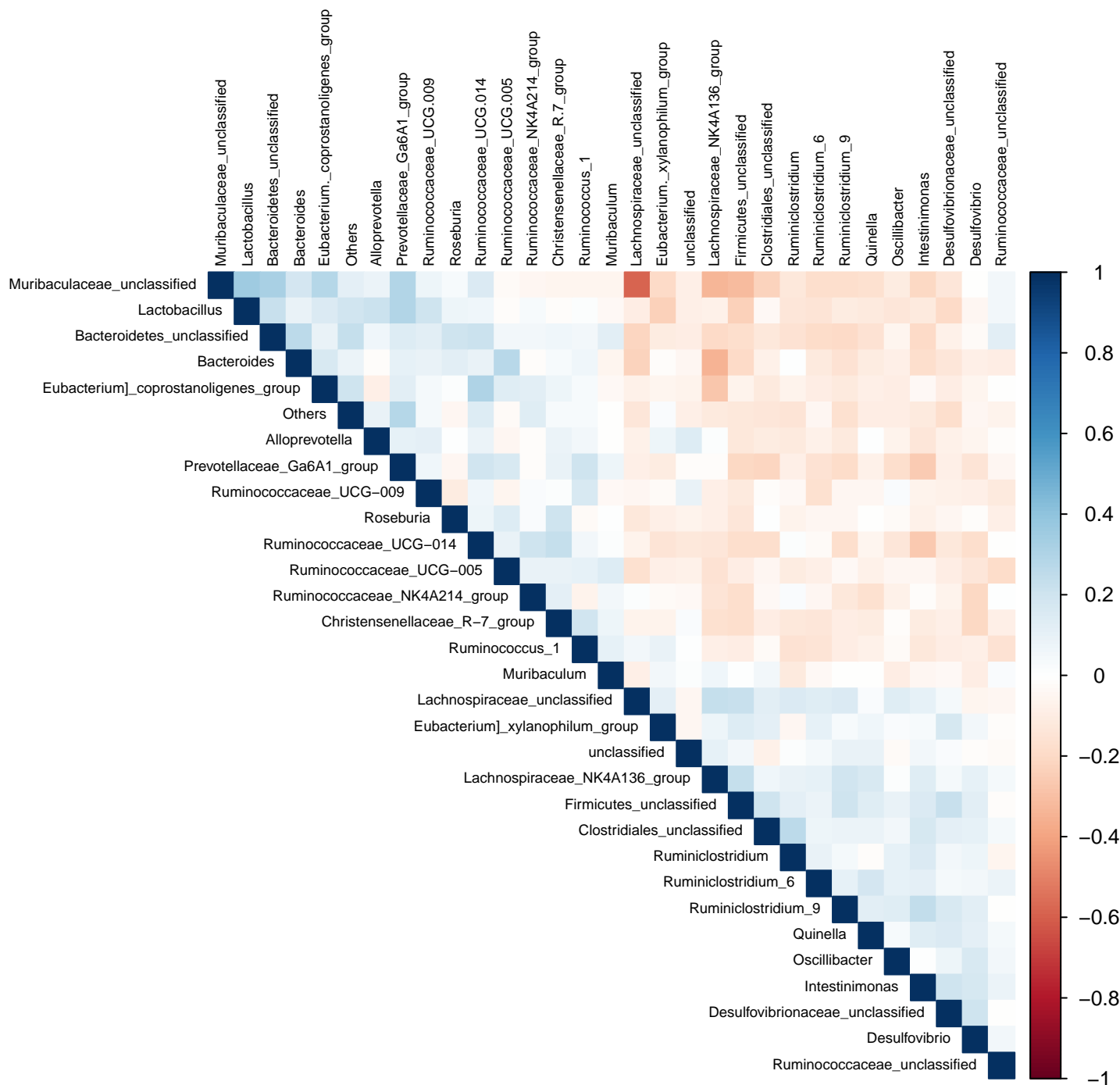

Supplement: Supplementary Materials — Supplementary Figure 1: genus phylotree of the intestinal flora of each group of rats. Supplementary Figure 2: interaction of the intestinal flora at the phylum level of Circos. Supplementary Figures 3 and 4 model group vs. normal group for differential species analysis. Supplementary Figures 5 and 6: BIFICO group vs. model group for differential species analysis. Supplementary Figures 7 and 8: LGL group vs. model group for differential species analysis. Supplementary Figures 9 and 10: LGH group vs. model group for differential species analysis. Supplementary Figure 11: based on random forest algorithm, the normal group was compared with the model group for feature species analysis. Supplementary Figure 12: based on random forest algorithm, the BIFICO group was compared with the model group for feature species analysis. Supplementary Figure 13: based on random forest algorithm, the LGL group was compared with the model group for feature species analysis. Supplementary Figure 14: based on random forest algorithm, the LGH group was compared with model group for feature species analysis. Supplementary Figure 15: correlation analysis of differential species. Supplementary Figure 16: redundancy analysis of flora at the phylum level. Supplementary Figure 17: redundancy analysis of flora at the genus level. Supplementary Figure 18: prediction of pathways for differential species function in the LGL group vs. model group. Supplementary Figure 19: prediction of pathways for differential species function in the LGH group vs. model group. Supplementary Figure 20: prediction of pathways based on annotation of the COG database on the function of differential species in the LGM group vs. model group. Supplementary Figure 21: prediction of pathways based on annotation of the EC database on the function of differential species in the LGL group vs. model group. Supplementary Figure 22: prediction of pathways based on annotation of the COG database on the function of differential spe [file 6256450.f1.zip › 6256450.f11.pdf]

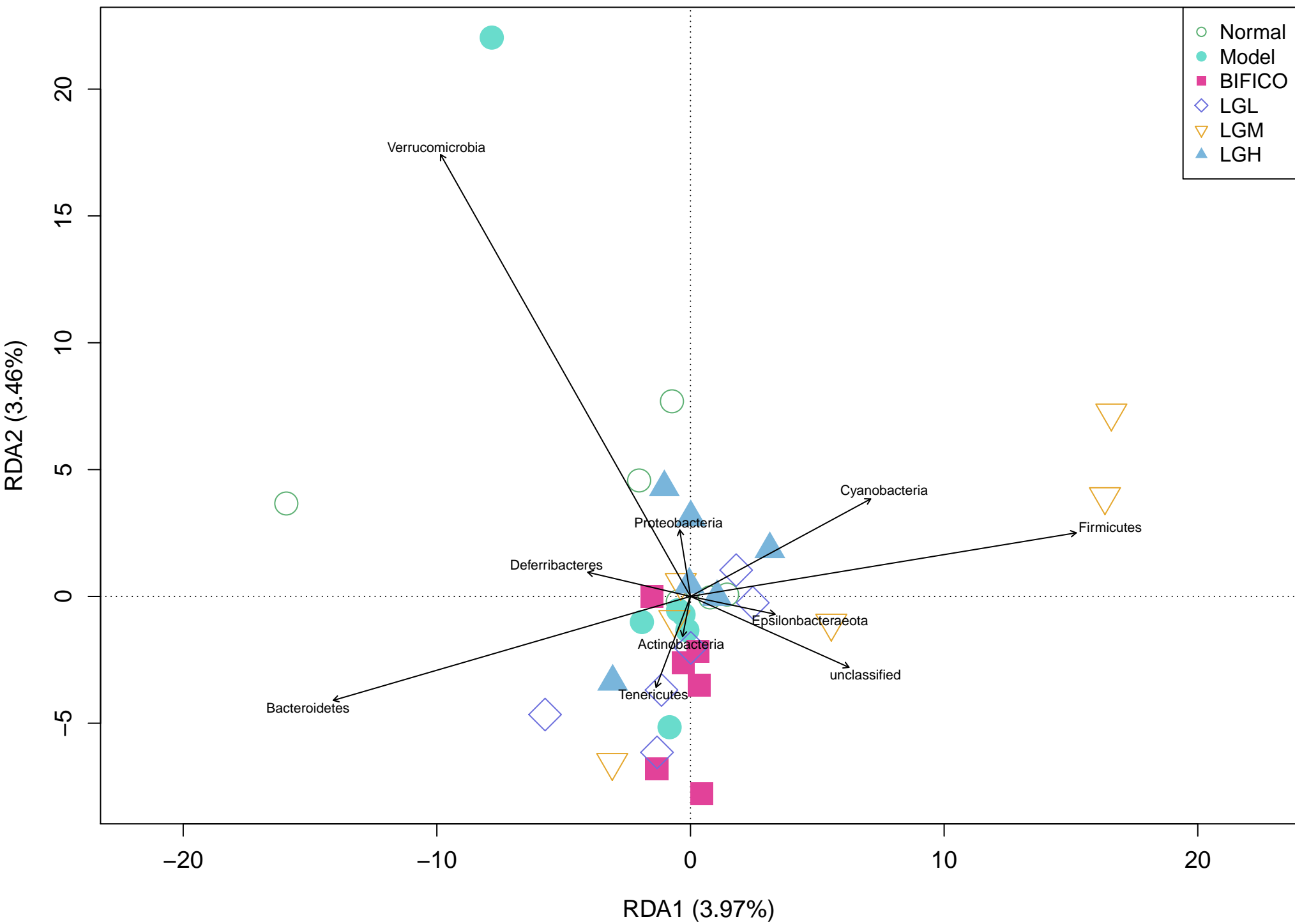

Supplement: Supplementary Materials — Supplementary Figure 1: genus phylotree of the intestinal flora of each group of rats. Supplementary Figure 2: interaction of the intestinal flora at the phylum level of Circos. Supplementary Figures 3 and 4 model group vs. normal group for differential species analysis. Supplementary Figures 5 and 6: BIFICO group vs. model group for differential species analysis. Supplementary Figures 7 and 8: LGL group vs. model group for differential species analysis. Supplementary Figures 9 and 10: LGH group vs. model group for differential species analysis. Supplementary Figure 11: based on random forest algorithm, the normal group was compared with the model group for feature species analysis. Supplementary Figure 12: based on random forest algorithm, the BIFICO group was compared with the model group for feature species analysis. Supplementary Figure 13: based on random forest algorithm, the LGL group was compared with the model group for feature species analysis. Supplementary Figure 14: based on random forest algorithm, the LGH group was compared with model group for feature species analysis. Supplementary Figure 15: correlation analysis of differential species. Supplementary Figure 16: redundancy analysis of flora at the phylum level. Supplementary Figure 17: redundancy analysis of flora at the genus level. Supplementary Figure 18: prediction of pathways for differential species function in the LGL group vs. model group. Supplementary Figure 19: prediction of pathways for differential species function in the LGH group vs. model group. Supplementary Figure 20: prediction of pathways based on annotation of the COG database on the function of differential species in the LGM group vs. model group. Supplementary Figure 21: prediction of pathways based on annotation of the EC database on the function of differential species in the LGL group vs. model group. Supplementary Figure 22: prediction of pathways based on annotation of the COG database on the function of differential spe [file 6256450.f1.zip › 6256450.f12.pdf]

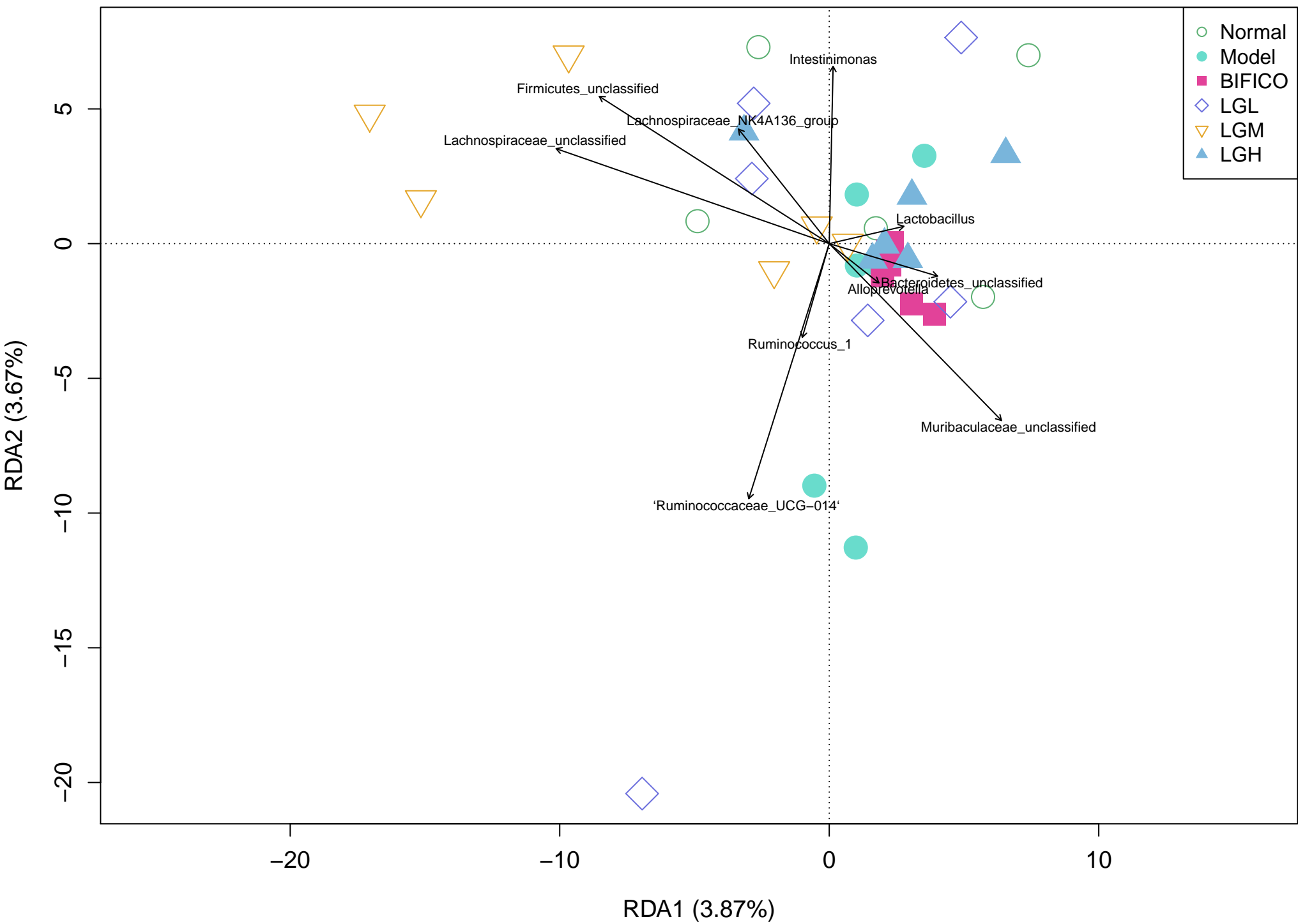

Supplement: Supplementary Materials — Supplementary Figure 1: genus phylotree of the intestinal flora of each group of rats. Supplementary Figure 2: interaction of the intestinal flora at the phylum level of Circos. Supplementary Figures 3 and 4 model group vs. normal group for differential species analysis. Supplementary Figures 5 and 6: BIFICO group vs. model group for differential species analysis. Supplementary Figures 7 and 8: LGL group vs. model group for differential species analysis. Supplementary Figures 9 and 10: LGH group vs. model group for differential species analysis. Supplementary Figure 11: based on random forest algorithm, the normal group was compared with the model group for feature species analysis. Supplementary Figure 12: based on random forest algorithm, the BIFICO group was compared with the model group for feature species analysis. Supplementary Figure 13: based on random forest algorithm, the LGL group was compared with the model group for feature species analysis. Supplementary Figure 14: based on random forest algorithm, the LGH group was compared with model group for feature species analysis. Supplementary Figure 15: correlation analysis of differential species. Supplementary Figure 16: redundancy analysis of flora at the phylum level. Supplementary Figure 17: redundancy analysis of flora at the genus level. Supplementary Figure 18: prediction of pathways for differential species function in the LGL group vs. model group. Supplementary Figure 19: prediction of pathways for differential species function in the LGH group vs. model group. Supplementary Figure 20: prediction of pathways based on annotation of the COG database on the function of differential species in the LGM group vs. model group. Supplementary Figure 21: prediction of pathways based on annotation of the EC database on the function of differential species in the LGL group vs. model group. Supplementary Figure 22: prediction of pathways based on annotation of the COG database on the function of differential spe [file 6256450.f1.zip › 6256450.f13.pdf]

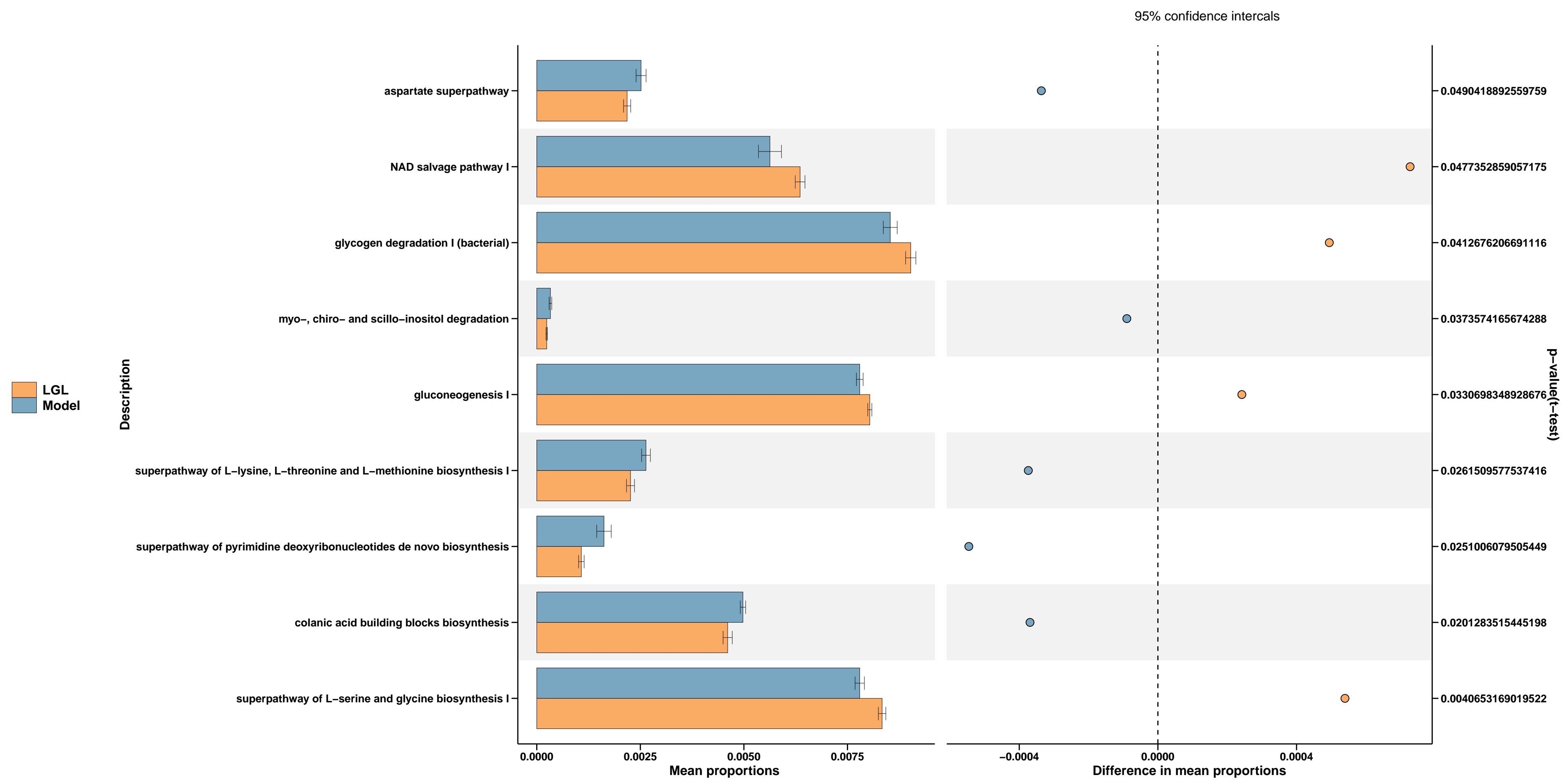

Supplement: Supplementary Materials — Supplementary Figure 1: genus phylotree of the intestinal flora of each group of rats. Supplementary Figure 2: interaction of the intestinal flora at the phylum level of Circos. Supplementary Figures 3 and 4 model group vs. normal group for differential species analysis. Supplementary Figures 5 and 6: BIFICO group vs. model group for differential species analysis. Supplementary Figures 7 and 8: LGL group vs. model group for differential species analysis. Supplementary Figures 9 and 10: LGH group vs. model group for differential species analysis. Supplementary Figure 11: based on random forest algorithm, the normal group was compared with the model group for feature species analysis. Supplementary Figure 12: based on random forest algorithm, the BIFICO group was compared with the model group for feature species analysis. Supplementary Figure 13: based on random forest algorithm, the LGL group was compared with the model group for feature species analysis. Supplementary Figure 14: based on random forest algorithm, the LGH group was compared with model group for feature species analysis. Supplementary Figure 15: correlation analysis of differential species. Supplementary Figure 16: redundancy analysis of flora at the phylum level. Supplementary Figure 17: redundancy analysis of flora at the genus level. Supplementary Figure 18: prediction of pathways for differential species function in the LGL group vs. model group. Supplementary Figure 19: prediction of pathways for differential species function in the LGH group vs. model group. Supplementary Figure 20: prediction of pathways based on annotation of the COG database on the function of differential species in the LGM group vs. model group. Supplementary Figure 21: prediction of pathways based on annotation of the EC database on the function of differential species in the LGL group vs. model group. Supplementary Figure 22: prediction of pathways based on annotation of the COG database on the function of differential spe [file 6256450.f1.zip › 6256450.f14.pdf]

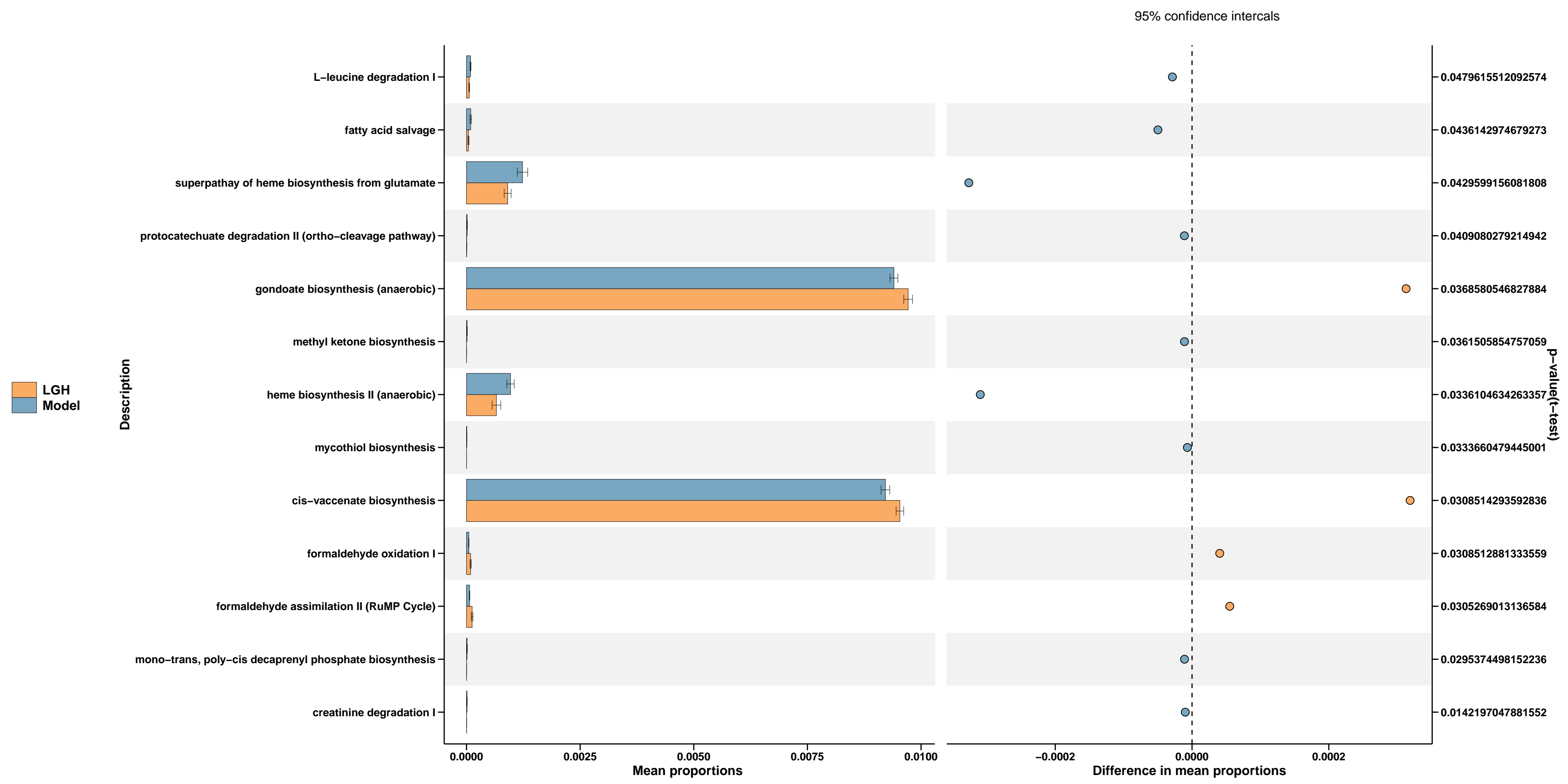

Supplement: Supplementary Materials — Supplementary Figure 1: genus phylotree of the intestinal flora of each group of rats. Supplementary Figure 2: interaction of the intestinal flora at the phylum level of Circos. Supplementary Figures 3 and 4 model group vs. normal group for differential species analysis. Supplementary Figures 5 and 6: BIFICO group vs. model group for differential species analysis. Supplementary Figures 7 and 8: LGL group vs. model group for differential species analysis. Supplementary Figures 9 and 10: LGH group vs. model group for differential species analysis. Supplementary Figure 11: based on random forest algorithm, the normal group was compared with the model group for feature species analysis. Supplementary Figure 12: based on random forest algorithm, the BIFICO group was compared with the model group for feature species analysis. Supplementary Figure 13: based on random forest algorithm, the LGL group was compared with the model group for feature species analysis. Supplementary Figure 14: based on random forest algorithm, the LGH group was compared with model group for feature species analysis. Supplementary Figure 15: correlation analysis of differential species. Supplementary Figure 16: redundancy analysis of flora at the phylum level. Supplementary Figure 17: redundancy analysis of flora at the genus level. Supplementary Figure 18: prediction of pathways for differential species function in the LGL group vs. model group. Supplementary Figure 19: prediction of pathways for differential species function in the LGH group vs. model group. Supplementary Figure 20: prediction of pathways based on annotation of the COG database on the function of differential species in the LGM group vs. model group. Supplementary Figure 21: prediction of pathways based on annotation of the EC database on the function of differential species in the LGL group vs. model group. Supplementary Figure 22: prediction of pathways based on annotation of the COG database on the function of differential spe [file 6256450.f1.zip › 6256450.f15.pdf]

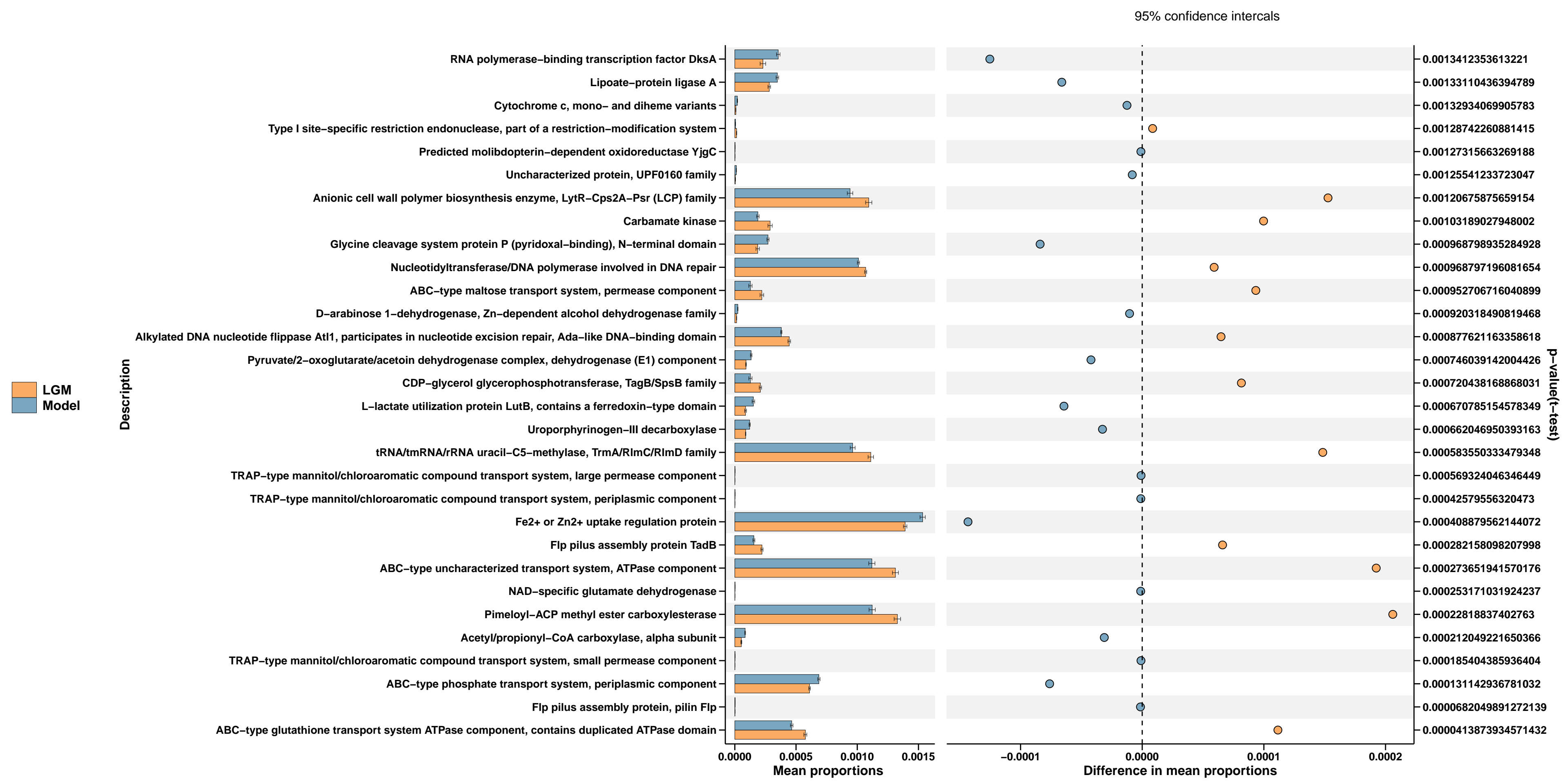

Supplement: Supplementary Materials — Supplementary Figure 1: genus phylotree of the intestinal flora of each group of rats. Supplementary Figure 2: interaction of the intestinal flora at the phylum level of Circos. Supplementary Figures 3 and 4 model group vs. normal group for differential species analysis. Supplementary Figures 5 and 6: BIFICO group vs. model group for differential species analysis. Supplementary Figures 7 and 8: LGL group vs. model group for differential species analysis. Supplementary Figures 9 and 10: LGH group vs. model group for differential species analysis. Supplementary Figure 11: based on random forest algorithm, the normal group was compared with the model group for feature species analysis. Supplementary Figure 12: based on random forest algorithm, the BIFICO group was compared with the model group for feature species analysis. Supplementary Figure 13: based on random forest algorithm, the LGL group was compared with the model group for feature species analysis. Supplementary Figure 14: based on random forest algorithm, the LGH group was compared with model group for feature species analysis. Supplementary Figure 15: correlation analysis of differential species. Supplementary Figure 16: redundancy analysis of flora at the phylum level. Supplementary Figure 17: redundancy analysis of flora at the genus level. Supplementary Figure 18: prediction of pathways for differential species function in the LGL group vs. model group. Supplementary Figure 19: prediction of pathways for differential species function in the LGH group vs. model group. Supplementary Figure 20: prediction of pathways based on annotation of the COG database on the function of differential species in the LGM group vs. model group. Supplementary Figure 21: prediction of pathways based on annotation of the EC database on the function of differential species in the LGL group vs. model group. Supplementary Figure 22: prediction of pathways based on annotation of the COG database on the function of differential spe [file 6256450.f1.zip › 6256450.f16.pdf]

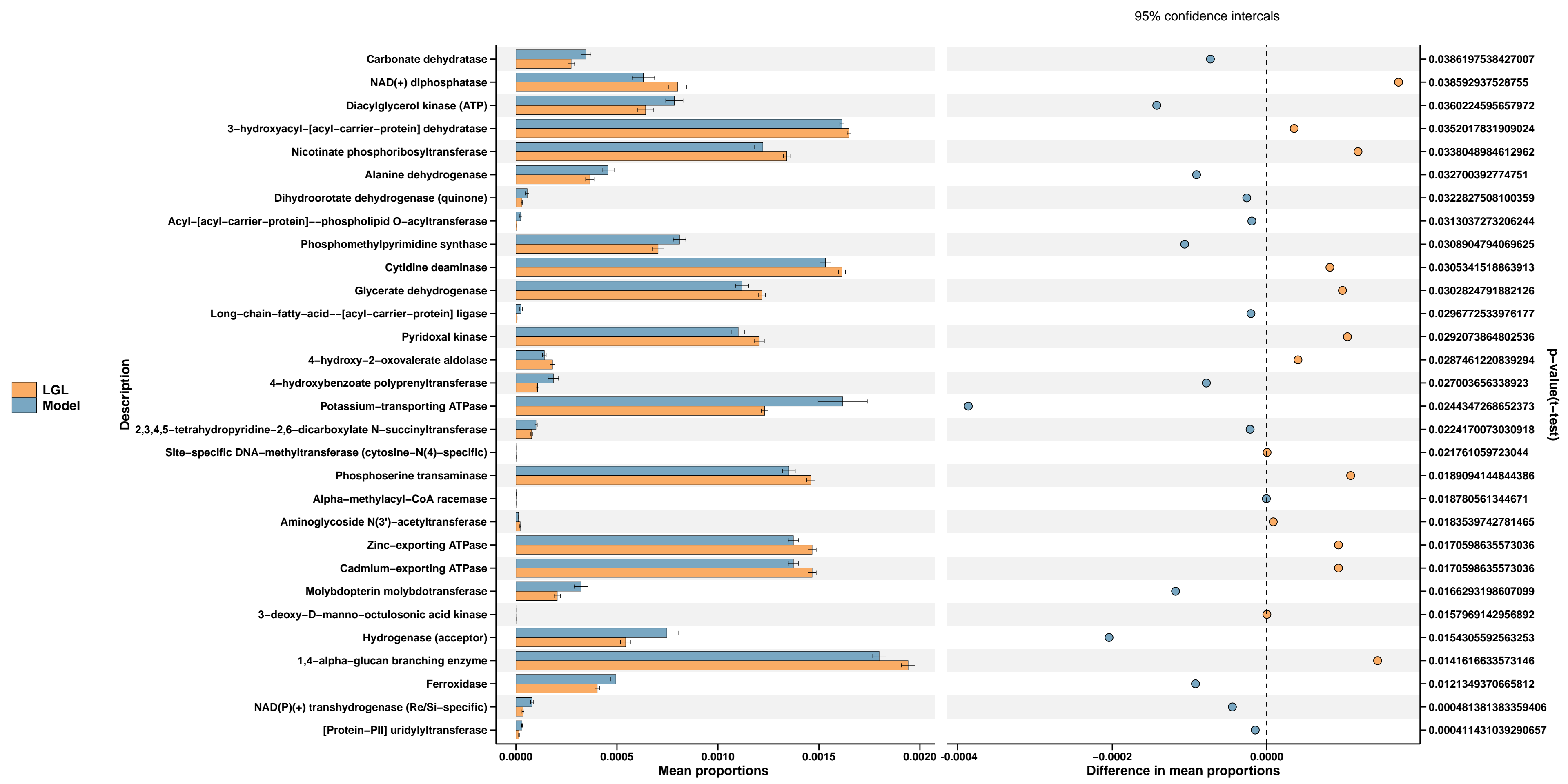

Supplement: Supplementary Materials — Supplementary Figure 1: genus phylotree of the intestinal flora of each group of rats. Supplementary Figure 2: interaction of the intestinal flora at the phylum level of Circos. Supplementary Figures 3 and 4 model group vs. normal group for differential species analysis. Supplementary Figures 5 and 6: BIFICO group vs. model group for differential species analysis. Supplementary Figures 7 and 8: LGL group vs. model group for differential species analysis. Supplementary Figures 9 and 10: LGH group vs. model group for differential species analysis. Supplementary Figure 11: based on random forest algorithm, the normal group was compared with the model group for feature species analysis. Supplementary Figure 12: based on random forest algorithm, the BIFICO group was compared with the model group for feature species analysis. Supplementary Figure 13: based on random forest algorithm, the LGL group was compared with the model group for feature species analysis. Supplementary Figure 14: based on random forest algorithm, the LGH group was compared with model group for feature species analysis. Supplementary Figure 15: correlation analysis of differential species. Supplementary Figure 16: redundancy analysis of flora at the phylum level. Supplementary Figure 17: redundancy analysis of flora at the genus level. Supplementary Figure 18: prediction of pathways for differential species function in the LGL group vs. model group. Supplementary Figure 19: prediction of pathways for differential species function in the LGH group vs. model group. Supplementary Figure 20: prediction of pathways based on annotation of the COG database on the function of differential species in the LGM group vs. model group. Supplementary Figure 21: prediction of pathways based on annotation of the EC database on the function of differential species in the LGL group vs. model group. Supplementary Figure 22: prediction of pathways based on annotation of the COG database on the function of differential spe [file 6256450.f1.zip › 6256450.f17.pdf]

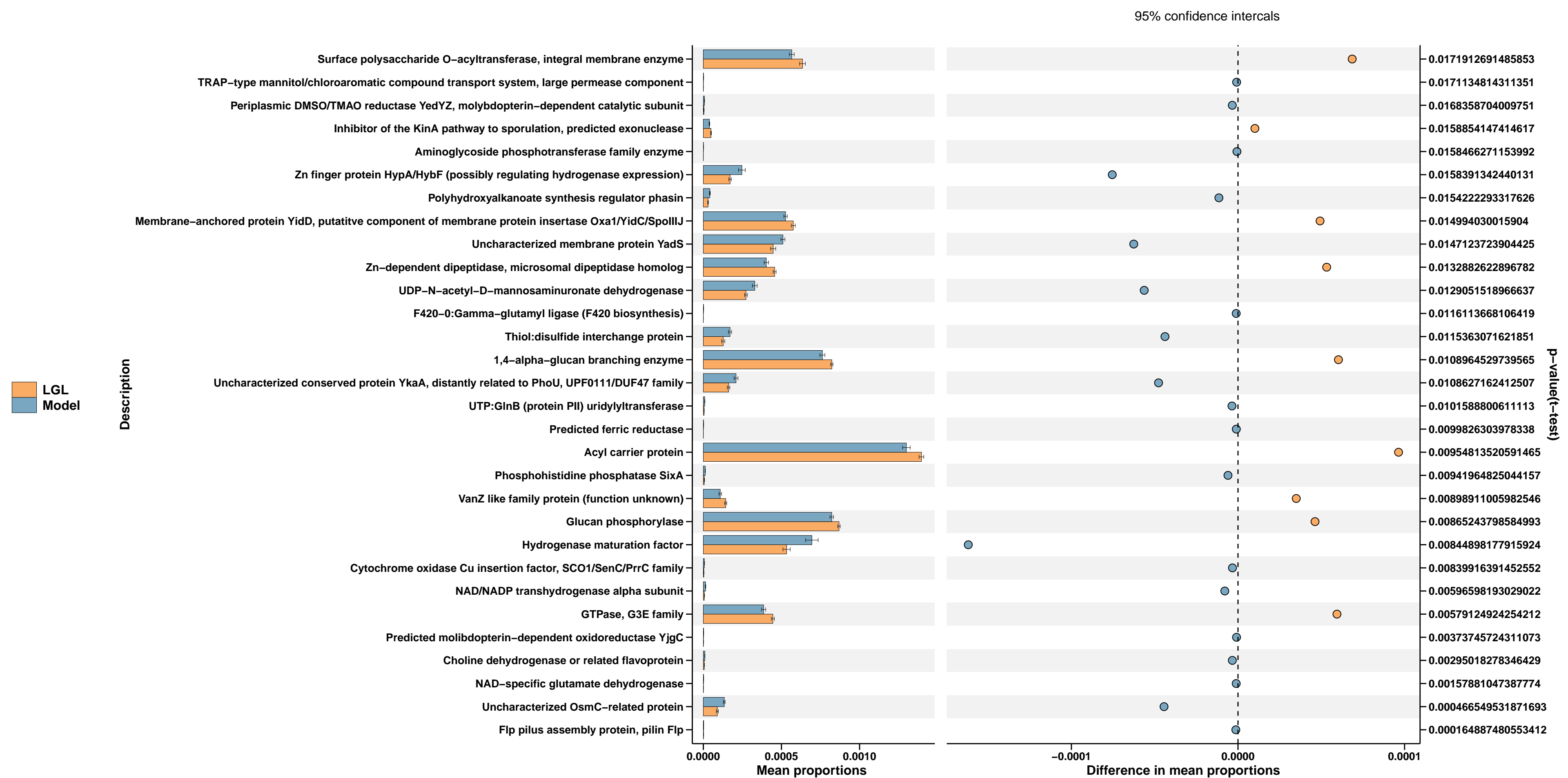

Supplement: Supplementary Materials — Supplementary Figure 1: genus phylotree of the intestinal flora of each group of rats. Supplementary Figure 2: interaction of the intestinal flora at the phylum level of Circos. Supplementary Figures 3 and 4 model group vs. normal group for differential species analysis. Supplementary Figures 5 and 6: BIFICO group vs. model group for differential species analysis. Supplementary Figures 7 and 8: LGL group vs. model group for differential species analysis. Supplementary Figures 9 and 10: LGH group vs. model group for differential species analysis. Supplementary Figure 11: based on random forest algorithm, the normal group was compared with the model group for feature species analysis. Supplementary Figure 12: based on random forest algorithm, the BIFICO group was compared with the model group for feature species analysis. Supplementary Figure 13: based on random forest algorithm, the LGL group was compared with the model group for feature species analysis. Supplementary Figure 14: based on random forest algorithm, the LGH group was compared with model group for feature species analysis. Supplementary Figure 15: correlation analysis of differential species. Supplementary Figure 16: redundancy analysis of flora at the phylum level. Supplementary Figure 17: redundancy analysis of flora at the genus level. Supplementary Figure 18: prediction of pathways for differential species function in the LGL group vs. model group. Supplementary Figure 19: prediction of pathways for differential species function in the LGH group vs. model group. Supplementary Figure 20: prediction of pathways based on annotation of the COG database on the function of differential species in the LGM group vs. model group. Supplementary Figure 21: prediction of pathways based on annotation of the EC database on the function of differential species in the LGL group vs. model group. Supplementary Figure 22: prediction of pathways based on annotation of the COG database on the function of differential spe [file 6256450.f1.zip › 6256450.f18.pdf]

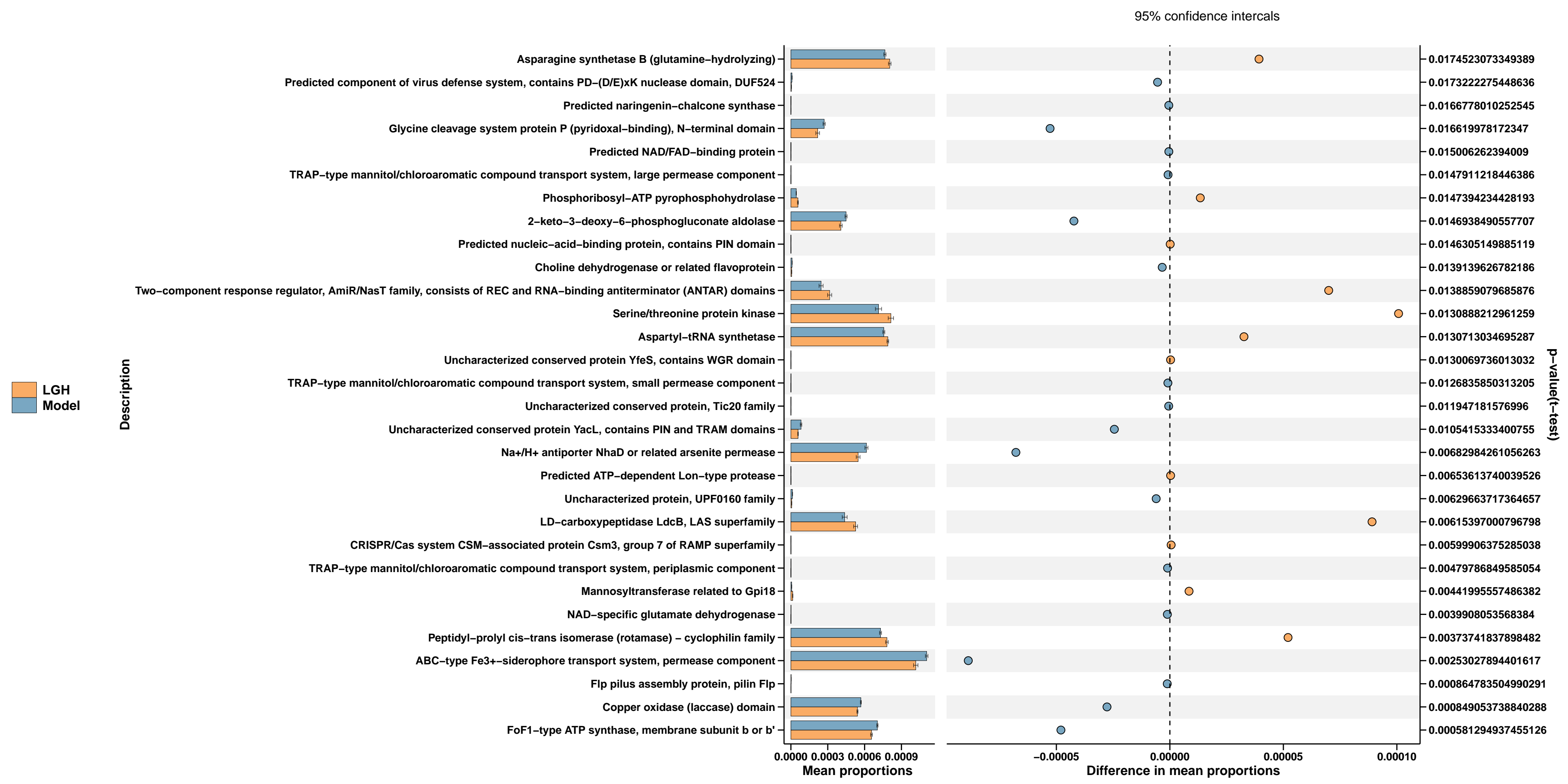

Supplement: Supplementary Materials — Supplementary Figure 1: genus phylotree of the intestinal flora of each group of rats. Supplementary Figure 2: interaction of the intestinal flora at the phylum level of Circos. Supplementary Figures 3 and 4 model group vs. normal group for differential species analysis. Supplementary Figures 5 and 6: BIFICO group vs. model group for differential species analysis. Supplementary Figures 7 and 8: LGL group vs. model group for differential species analysis. Supplementary Figures 9 and 10: LGH group vs. model group for differential species analysis. Supplementary Figure 11: based on random forest algorithm, the normal group was compared with the model group for feature species analysis. Supplementary Figure 12: based on random forest algorithm, the BIFICO group was compared with the model group for feature species analysis. Supplementary Figure 13: based on random forest algorithm, the LGL group was compared with the model group for feature species analysis. Supplementary Figure 14: based on random forest algorithm, the LGH group was compared with model group for feature species analysis. Supplementary Figure 15: correlation analysis of differential species. Supplementary Figure 16: redundancy analysis of flora at the phylum level. Supplementary Figure 17: redundancy analysis of flora at the genus level. Supplementary Figure 18: prediction of pathways for differential species function in the LGL group vs. model group. Supplementary Figure 19: prediction of pathways for differential species function in the LGH group vs. model group. Supplementary Figure 20: prediction of pathways based on annotation of the COG database on the function of differential species in the LGM group vs. model group. Supplementary Figure 21: prediction of pathways based on annotation of the EC database on the function of differential species in the LGL group vs. model group. Supplementary Figure 22: prediction of pathways based on annotation of the COG database on the function of differential spe [file 6256450.f1.zip › 6256450.f19.pdf]

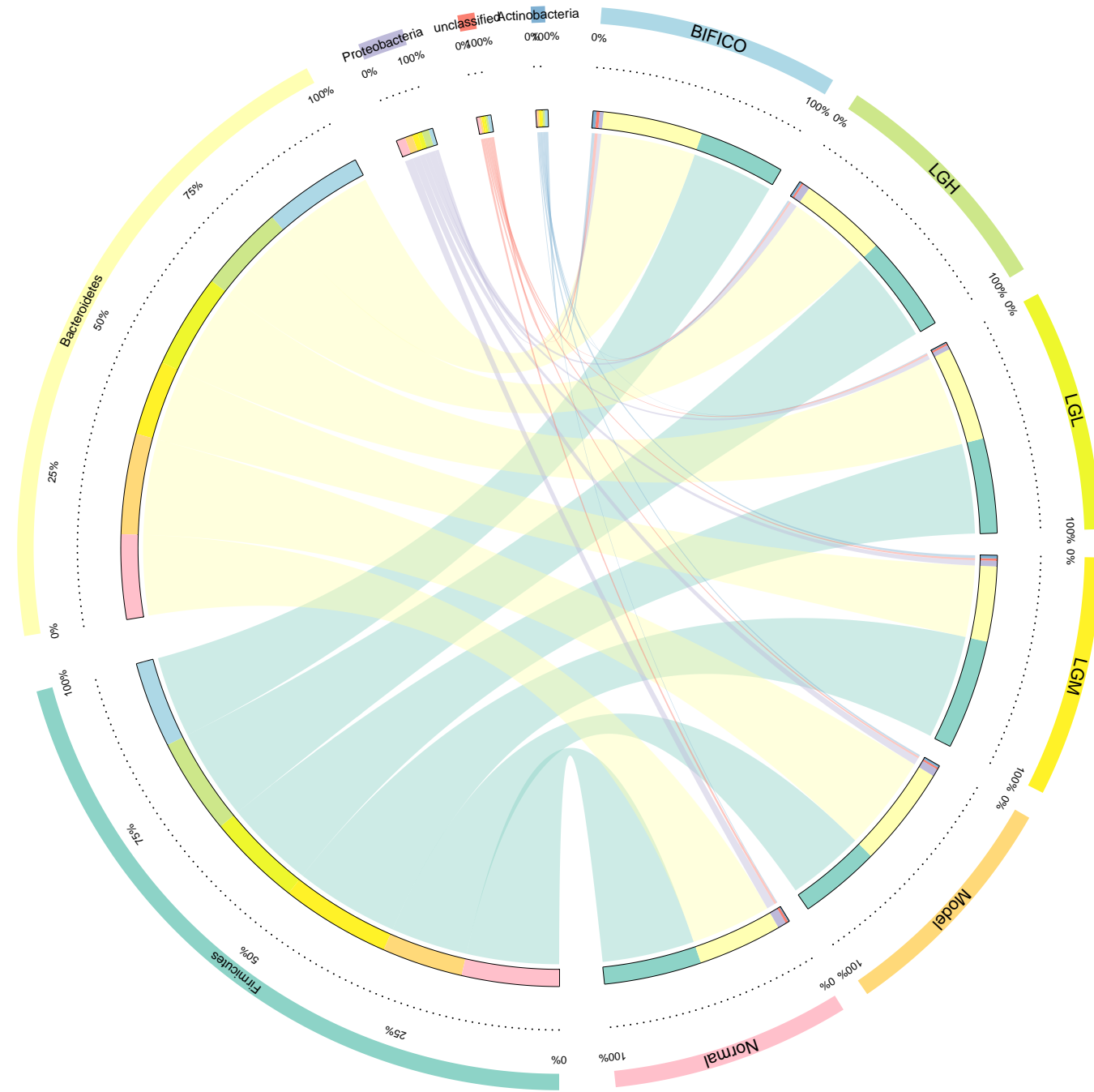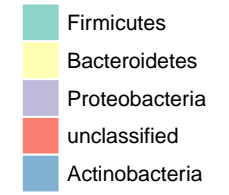

Supplement: Supplementary Materials — Supplementary Figure 1: genus phylotree of the intestinal flora of each group of rats. Supplementary Figure 2: interaction of the intestinal flora at the phylum level of Circos. Supplementary Figures 3 and 4 model group vs. normal group for differential species analysis. Supplementary Figures 5 and 6: BIFICO group vs. model group for differential species analysis. Supplementary Figures 7 and 8: LGL group vs. model group for differential species analysis. Supplementary Figures 9 and 10: LGH group vs. model group for differential species analysis. Supplementary Figure 11: based on random forest algorithm, the normal group was compared with the model group for feature species analysis. Supplementary Figure 12: based on random forest algorithm, the BIFICO group was compared with the model group for feature species analysis. Supplementary Figure 13: based on random forest algorithm, the LGL group was compared with the model group for feature species analysis. Supplementary Figure 14: based on random forest algorithm, the LGH group was compared with model group for feature species analysis. Supplementary Figure 15: correlation analysis of differential species. Supplementary Figure 16: redundancy analysis of flora at the phylum level. Supplementary Figure 17: redundancy analysis of flora at the genus level. Supplementary Figure 18: prediction of pathways for differential species function in the LGL group vs. model group. Supplementary Figure 19: prediction of pathways for differential species function in the LGH group vs. model group. Supplementary Figure 20: prediction of pathways based on annotation of the COG database on the function of differential species in the LGM group vs. model group. Supplementary Figure 21: prediction of pathways based on annotation of the EC database on the function of differential species in the LGL group vs. model group. Supplementary Figure 22: prediction of pathways based on annotation of the COG database on the function of differential spe [file 6256450.f1.zip › 6256450.f2.pdf]

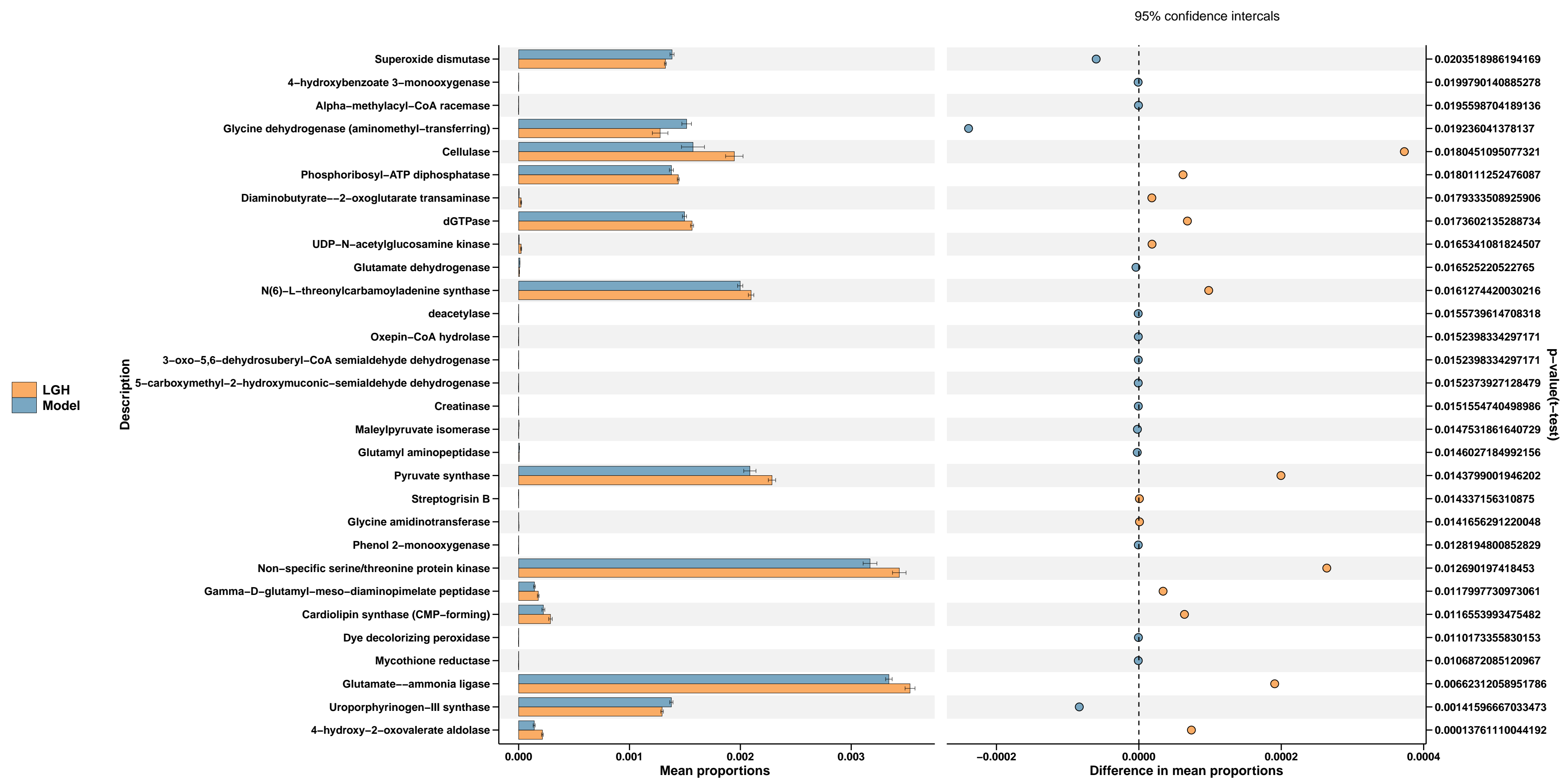

Supplement: Supplementary Materials — Supplementary Figure 1: genus phylotree of the intestinal flora of each group of rats. Supplementary Figure 2: interaction of the intestinal flora at the phylum level of Circos. Supplementary Figures 3 and 4 model group vs. normal group for differential species analysis. Supplementary Figures 5 and 6: BIFICO group vs. model group for differential species analysis. Supplementary Figures 7 and 8: LGL group vs. model group for differential species analysis. Supplementary Figures 9 and 10: LGH group vs. model group for differential species analysis. Supplementary Figure 11: based on random forest algorithm, the normal group was compared with the model group for feature species analysis. Supplementary Figure 12: based on random forest algorithm, the BIFICO group was compared with the model group for feature species analysis. Supplementary Figure 13: based on random forest algorithm, the LGL group was compared with the model group for feature species analysis. Supplementary Figure 14: based on random forest algorithm, the LGH group was compared with model group for feature species analysis. Supplementary Figure 15: correlation analysis of differential species. Supplementary Figure 16: redundancy analysis of flora at the phylum level. Supplementary Figure 17: redundancy analysis of flora at the genus level. Supplementary Figure 18: prediction of pathways for differential species function in the LGL group vs. model group. Supplementary Figure 19: prediction of pathways for differential species function in the LGH group vs. model group. Supplementary Figure 20: prediction of pathways based on annotation of the COG database on the function of differential species in the LGM group vs. model group. Supplementary Figure 21: prediction of pathways based on annotation of the EC database on the function of differential species in the LGL group vs. model group. Supplementary Figure 22: prediction of pathways based on annotation of the COG database on the function of differential spe [file 6256450.f1.zip › 6256450.f20.pdf]

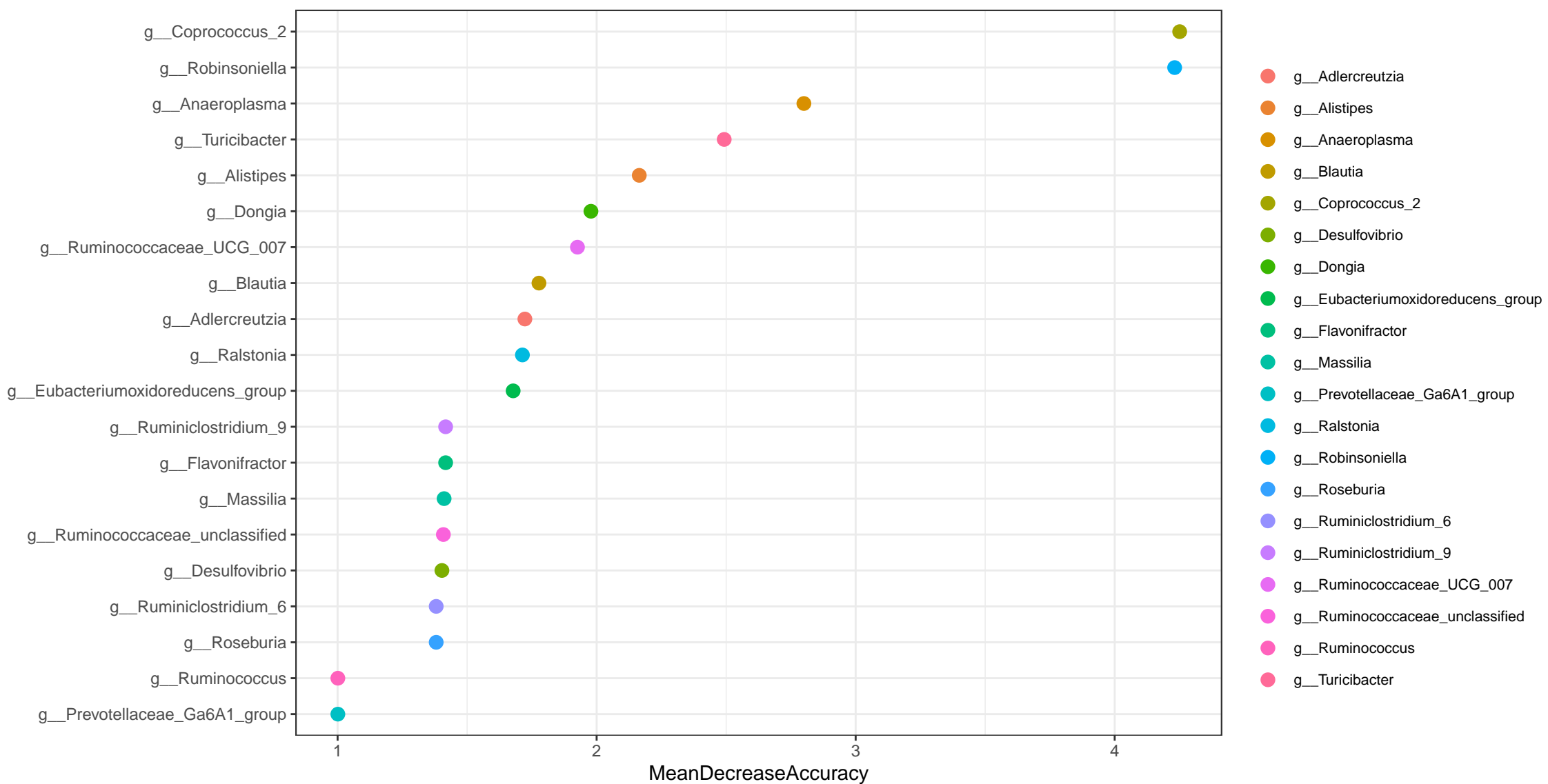

Supplement: Supplementary Materials — Supplementary Figure 1: genus phylotree of the intestinal flora of each group of rats. Supplementary Figure 2: interaction of the intestinal flora at the phylum level of Circos. Supplementary Figures 3 and 4 model group vs. normal group for differential species analysis. Supplementary Figures 5 and 6: BIFICO group vs. model group for differential species analysis. Supplementary Figures 7 and 8: LGL group vs. model group for differential species analysis. Supplementary Figures 9 and 10: LGH group vs. model group for differential species analysis. Supplementary Figure 11: based on random forest algorithm, the normal group was compared with the model group for feature species analysis. Supplementary Figure 12: based on random forest algorithm, the BIFICO group was compared with the model group for feature species analysis. Supplementary Figure 13: based on random forest algorithm, the LGL group was compared with the model group for feature species analysis. Supplementary Figure 14: based on random forest algorithm, the LGH group was compared with model group for feature species analysis. Supplementary Figure 15: correlation analysis of differential species. Supplementary Figure 16: redundancy analysis of flora at the phylum level. Supplementary Figure 17: redundancy analysis of flora at the genus level. Supplementary Figure 18: prediction of pathways for differential species function in the LGL group vs. model group. Supplementary Figure 19: prediction of pathways for differential species function in the LGH group vs. model group. Supplementary Figure 20: prediction of pathways based on annotation of the COG database on the function of differential species in the LGM group vs. model group. Supplementary Figure 21: prediction of pathways based on annotation of the EC database on the function of differential species in the LGL group vs. model group. Supplementary Figure 22: prediction of pathways based on annotation of the COG database on the function of differential spe [file 6256450.f1.zip › 6256450.f7.pdf]

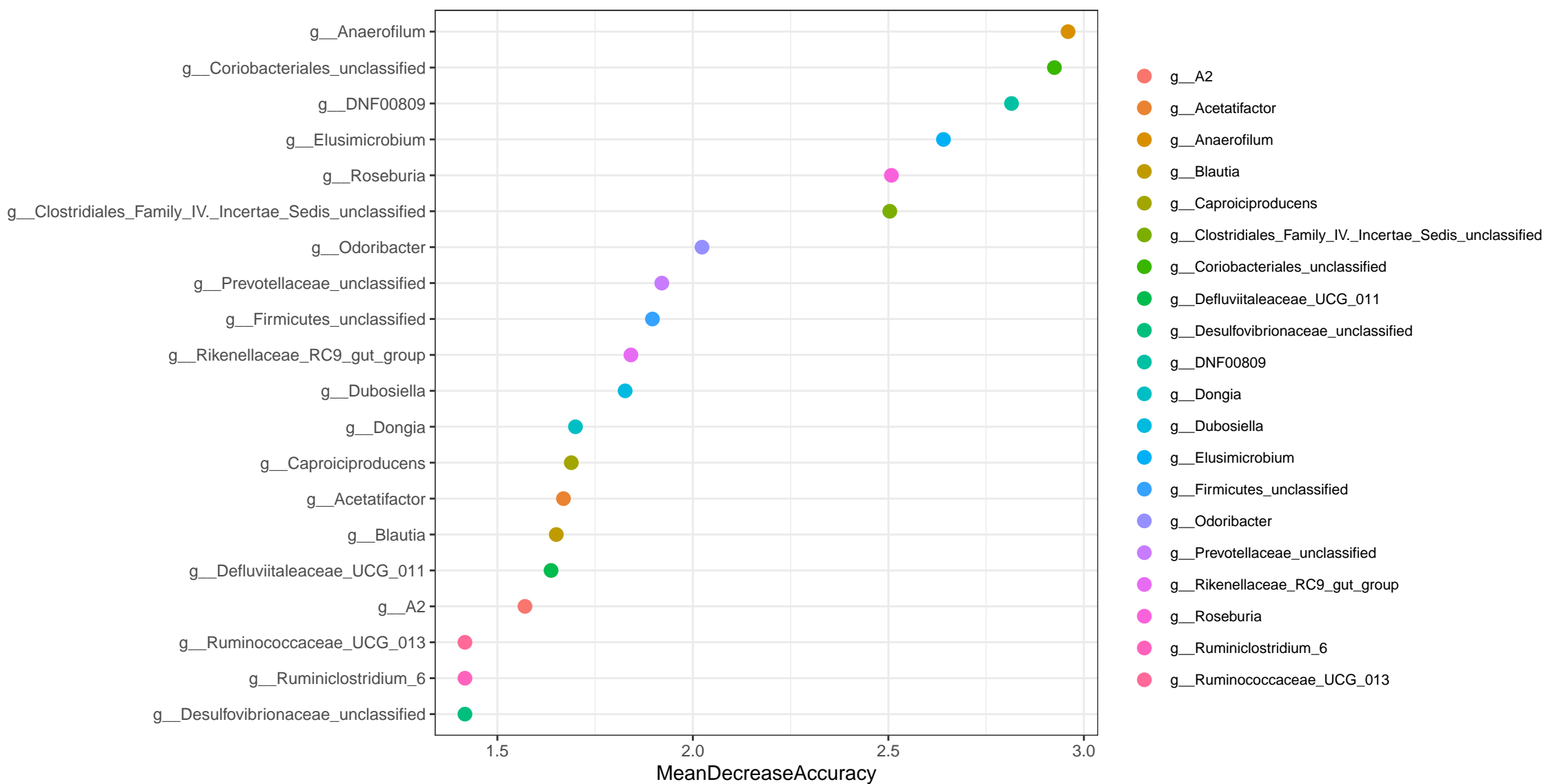

Supplement: Supplementary Materials — Supplementary Figure 1: genus phylotree of the intestinal flora of each group of rats. Supplementary Figure 2: interaction of the intestinal flora at the phylum level of Circos. Supplementary Figures 3 and 4 model group vs. normal group for differential species analysis. Supplementary Figures 5 and 6: BIFICO group vs. model group for differential species analysis. Supplementary Figures 7 and 8: LGL group vs. model group for differential species analysis. Supplementary Figures 9 and 10: LGH group vs. model group for differential species analysis. Supplementary Figure 11: based on random forest algorithm, the normal group was compared with the model group for feature species analysis. Supplementary Figure 12: based on random forest algorithm, the BIFICO group was compared with the model group for feature species analysis. Supplementary Figure 13: based on random forest algorithm, the LGL group was compared with the model group for feature species analysis. Supplementary Figure 14: based on random forest algorithm, the LGH group was compared with model group for feature species analysis. Supplementary Figure 15: correlation analysis of differential species. Supplementary Figure 16: redundancy analysis of flora at the phylum level. Supplementary Figure 17: redundancy analysis of flora at the genus level. Supplementary Figure 18: prediction of pathways for differential species function in the LGL group vs. model group. Supplementary Figure 19: prediction of pathways for differential species function in the LGH group vs. model group. Supplementary Figure 20: prediction of pathways based on annotation of the COG database on the function of differential species in the LGM group vs. model group. Supplementary Figure 21: prediction of pathways based on annotation of the EC database on the function of differential species in the LGL group vs. model group. Supplementary Figure 22: prediction of pathways based on annotation of the COG database on the function of differential spe [file 6256450.f1.zip › 6256450.f8.pdf]

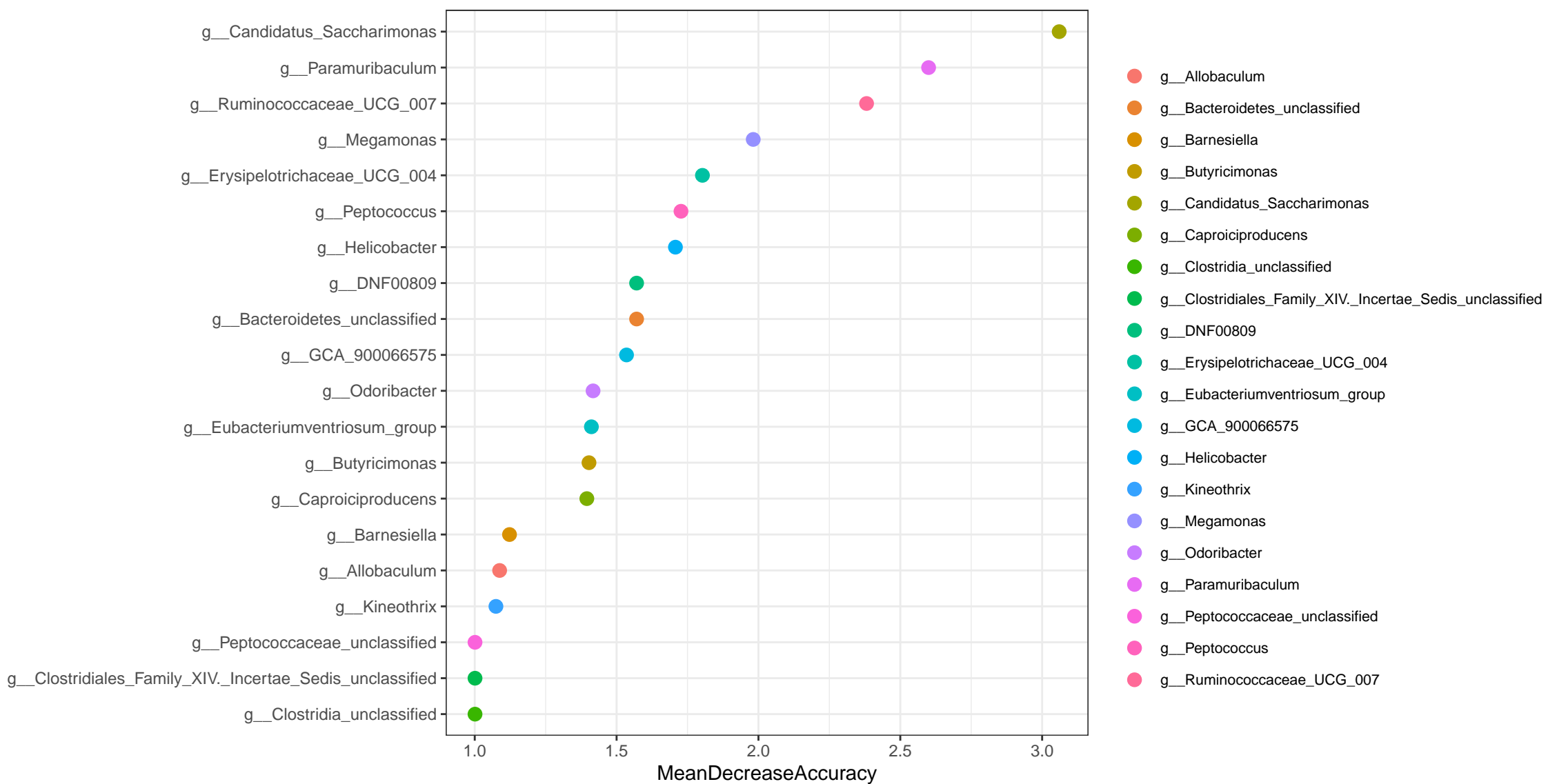

Supplement: Supplementary Materials — Supplementary Figure 1: genus phylotree of the intestinal flora of each group of rats. Supplementary Figure 2: interaction of the intestinal flora at the phylum level of Circos. Supplementary Figures 3 and 4 model group vs. normal group for differential species analysis. Supplementary Figures 5 and 6: BIFICO group vs. model group for differential species analysis. Supplementary Figures 7 and 8: LGL group vs. model group for differential species analysis. Supplementary Figures 9 and 10: LGH group vs. model group for differential species analysis. Supplementary Figure 11: based on random forest algorithm, the normal group was compared with the model group for feature species analysis. Supplementary Figure 12: based on random forest algorithm, the BIFICO group was compared with the model group for feature species analysis. Supplementary Figure 13: based on random forest algorithm, the LGL group was compared with the model group for feature species analysis. Supplementary Figure 14: based on random forest algorithm, the LGH group was compared with model group for feature species analysis. Supplementary Figure 15: correlation analysis of differential species. Supplementary Figure 16: redundancy analysis of flora at the phylum level. Supplementary Figure 17: redundancy analysis of flora at the genus level. Supplementary Figure 18: prediction of pathways for differential species function in the LGL group vs. model group. Supplementary Figure 19: prediction of pathways for differential species function in the LGH group vs. model group. Supplementary Figure 20: prediction of pathways based on annotation of the COG database on the function of differential species in the LGM group vs. model group. Supplementary Figure 21: prediction of pathways based on annotation of the EC database on the function of differential species in the LGL group vs. model group. Supplementary Figure 22: prediction of pathways based on annotation of the COG database on the function of differential spe [file 6256450.f1.zip › 6256450.f9.pdf]
